# Severe subcutaneous infection with *Clostridium septicum* in a herd of native Icelandic horses

**DOI:** 10.1186/s13028-025-00792-y

**Published:** 2025-02-06

**Authors:** Charlotta Oddsdóttir, Ólöf G. Sigurðardóttir, Vala Friðriksdóttir, Vilhjálmur Svansson, Birkir Þór Bragason, Sigríður Björnsdóttir

**Affiliations:** 1 Division of Bacteriology and Pathology, Institute for Experimental Pathology at Keldur, Keldnavegi 3, 112 Reykjavík, Iceland; 2 Division of Virology, Molecular Biology and Parasitology, Institute for Experimental Pathology at Keldur, Keldnavegi 3, 112 Reykjavík, Iceland; 3 Icelandic Food and Veterinary Authority, Austurvegi 64, 800 Selfoss, Iceland

**Keywords:** Anthelmintic, Cellulitis, Clostridia, Equine, Fatal, Genome sequencing, Injection, Ivermectin, Outbreak

## Abstract

**Background:**

Cellulitis due to infection with clostridia has not been documented in horses in Iceland. However, clostridia are well-known pathogens in Icelandic sheep, which have traditionally shared grazing land with horses. Clostridial infections of equine muscle or subcutis following injection with medicinal products have been described in other countries but have never been reported in Iceland. In this case report, we present the first documented outbreak of subcutaneous clostridial infection in horses in Iceland following subcutaneous injection.

**Case presentation:**

In November 2022, 16 out of 32 horses, that some days earlier had received a subcutaneous injection of Noromectin^®^ 1% injectable solution, developed clinical signs indicating malignant oedema. The clinical signs included pyrexia, depression, swollen limbs, chest and neck, reluctance to move and dyspnoea, leading to the death or euthanasia of five horses. In addition, one horse was found dead with no previously noted clinical signs. Necropsy of one of the five horses revealed severe, acute cellulitis in the neck region, as well as lymphadenitis in regional lymph nodes. Abscesses, some with subsequent spontaneous drainage of seropurulent material, were observed at the presumed injection site on eight surviving horses approximately 2 weeks post-injection. Bacterial culture of samples from the necropsied horse and from abscesses from three surviving horses yielded the growth of *C. septicum*. Analysis of water samples from the pasture where the herd was kept also revealed the presence of *C. septicum*. Whole genome sequencing suggested that the isolates from the diseased horses contained the same *C. septicum* strain, whereas the strain isolated from the water samples differed from the disease-causing isolates.

**Conclusions:**

Clinical signs compatible with serious subcutaneous *C.* *septicum* infection were seen in over half of 32 horses injected with an ivermectin product, with the subsequent death of six of the horses. In the absence of other obvious sources, the outbreak suggests that *C. septicum* spores on the skin of these horses were introduced under the skin when they were injected. Such infections have not been reported in Iceland, although ivermectin products formulated for subcutaneous injection have been widely used for more than 30 years. The outbreak warrants further investigation into *C. septicum* in the environment of grazing horses in Iceland.

**Supplementary Information:**

The online version contains supplementary material available at 10.1186/s13028-025-00792-y.

## Background

Clostridial diseases are known to occur in domestic animals in Iceland, and many sheep herds are vaccinated against the toxins produced by *Clostridium perfringens* and *Clostridium septicum*, which causes braxy. Diseases caused by clostridia are rarely diagnosed in horses in Iceland, except for the occasional outbreak of botulism due to the ingestion of haylage contaminated with *Clostridium botulinum* toxin [1, 2]. In other countries, clostridial infections following subcutaneous or intramuscular injections have been reported in sheep [3], cattle [4] and horses [5, 6]. In Iceland, there are only sporadic tetanus outbreaks due to *Clostridium tetani* in sheep and horses.

There is no mandatory vaccination programme for horses in Iceland due to the absence of many infectious equine diseases and the sporadic nature of clostridial enterotoxaemia. Horses are commonly kept grazing in free-roaming herds, and in winter, are fed haylage. This type of extensive management method is most common for breeding mares with foals, youngsters, and adult riding horses that have been given time off.

For more than 30 years, preventive herd treatment with subcutaneous ivermectin medication has been common practice during the autumn months [7]. In addition to a rather broad antiparasitic effect, a preventive effect against biting lice (*Werneckiella (Damalinia) equi*) has been assumed. Although practitioners are aware of sporadic, local reactions to the product and a risk of infection associated with breaking potentially contaminated skin, the risk has generally been deemed to be acceptable. However, in late November 2022, an outbreak of disease occurred in a group of 32 horses. The horses were kept by the same manager in a free-roaming herd and stable. All of the horses received a subcutaneous injection with Noromectin^®^ 1% injectable solution, an off-label ivermectin product, and half of them showed severe clinical signs in the days and weeks post-injection. In addition, one horse was found dead with no previously noted clinical signs. *C. septicum* was isolated in almost pure culture from the abscesses of three surviving horses and from the material sampled at necropsy of one horse. In addition to one sudden death, 16 out of 32 horses developed clinical signs over the course of 2 weeks, with three horses dying and two being euthanised. To the best of the authors’ knowledge, this study reports the first documented outbreak of subcutaneous clostridial infection following subcutaneous injection of horses in Iceland, and indeed the Nordic countries.

## Case presentation

On 25 November 2022, a practising veterinarian notified the Icelandic Veterinary Authority of an illness in horses originating from or having been in contact with a herd on pasture in the South of Iceland. In total, 32 horses varying in age from a few months to 21 years were involved (Table 1). The animals had received subcutaneous ivermectin injections (Noromectin^®^ 1% solution, Norbrook Laboratories Limited, Co. Down, NI); the 28 free-roaming horses on 21 November, and four stabled horses on 23 November. The weather had been unusually warm (around 5 °C) and wet for the season (around 130 mm of rain during the month of November, with 33 mm rainfall on 14 November) and the 30-ha pasture where most of the horses were kept was very wet, with puddles and larger accumulations of stagnant water. Five pregnant mares and their foals had been kept in a smaller, drier pasture of 7-ha, but were reunited with the rest of the herd in the 30-ha pasture on 21 November after receiving the ivermectin injections. In addition to the 32 horses injected, the owners had not managed to inject one of the five foals, which evaded being caught. None of these foals developed clinical signs, neither the ones injected, nor the one not injected.

**Table 1:** Overview of a herd of 32 Icelandic horses given subcutaneous ivermectin injections

Horse No	Age in years	Injection date	Early clinical signs	Date of first signs	Later development and date of observation
1	9	21 Nov	Pyrexia, oedema, stiffness	23 Nov	
2	7	21 Nov	Pyrexia, oedema, stiffness	23 Nov	Broken skin on chest, 5 Dec
3	4	21 Nov	Pyrexia	24 Nov	Fistulation of abscess, 9 Dec
4	5	23 Nov	Pyrexia, oedema	25 Nov	Swelling on neck, 5 Dec
5	5	21 Nov	Pyrexia	25 Nov	Fistulation of abscess, 9 Dec
6	22	21 Nov	Pyrexia, oedema	25 Nov	Dyspnoea, euthanasia, 26 Nov
7	14	21 Nov	Pyrexia	25 Nov	Death, 27 Nov
8	5	21 Nov	Pyrexia, oedema, stiffness	25 Nov	Death, 28 Nov
9	4	21 Nov	Pyrexia, oedema, stiffness	25 Nov	Death, 28 Nov
10	6	21 Nov	Pyrexia, oedema	25 Nov	Dyspnoea, euthanasia, 30 Nov
11	4	21 Nov	Pyrexia	25 Nov	
12	3	21 Nov	Found dead, no preceding clinical signs	26 Nov	
13	5	23 Nov	Pyrexia	27 Nov	Swelling on neck, 5 Dec
14	11	23 Nov	–		Swelling on neck, 5 Dec
15	5	23 Nov	–		Swelling on neck, 5 Dec
16	2	21 Nov	–		Fistulation of abscess, 9 Dec
17	0.5	21 Nov	–		Fistulation of abscess, 9 Dec
18	20	21 Nov	–		
19	20	21 Nov	–		
20	17	21 Nov	–		
21	16	21 Nov	–		
22	15	21 Nov	–		
23	8	21 Nov	–		
24	5	21 Nov	–		
25	4	21 Nov	–		
26	3	21 Nov	–		
27	3	21 Nov	–		
28	3	21 Nov	–		
29	0.5	21 Nov	–		
30	0.5	21 Nov	–		
31	0.5	21 Nov	–		
32	0.5	21 Nov	–		

List of horses that received subcutaneous ivermectin injections on 21 and 23 November 2022 and the clinical signs recorded in 16 out of these 32 horses in the days that followed

One horse (Horse 12) was found dead without any clinical signs having been recorded. Six horses in the herd died in the space of five days, and eight surviving horses developed abscesses/swellings on the neck at the injection site approximately two weeks post-injection

The horses are numbered 1–32; no clinical signs were noted in horses 18-32 during the investigation period

The table shows the age (in years) of each horse (age set at 0.5 years for foals born in the summer 2022), as well as early clinical signs and the development of clinical signs over the two weeks post-injection

-: no clinical signs observed

On 23 November, the owners noticed that two stabled horses (Horses 1 and 2, Table 1) were reluctant to move, exhibiting stiff gait. Both horses had been treated subcutaneously with ivermectin in the herd on 21 November. Over the next few days, these horses developed pyrexia (with a rectal temperature of 40 °C), depression and swelling of the forelimbs. On 25 November, an additional nine horses (Horses 3–11) were found with similar signs (with rectal temperatures of 38.7–41 °C). Of these horses, eight were from the free-range herd, but one (Horse 4) was stabled and had received an ivermectin injection on 23 November. On that same day, blood samples, nasal swabs and faecal samples were collected from the stabled horses for initial virological diagnostics.

The first fatality occurred on 26 November, when a three-year-old gelding (Horse 12) was found dead without any observed previous clinical signs. On the same day, a twenty-two-year-old gelding (Horse 6) was euthanised for welfare reasons due to severe breathing difficulties. On 27 November, a fourteen-year-old pregnant mare (Horse 7) was found dead on pasture following 2 days of pyrexia. On 28 November, two mares of four and five years old (Horses 8 and 9) died after having fever and swollen limbs the day before. One of these (Horse 8) was subjected to a field postmortem examination with the tentative diagnosis of salmonellosis. Samples of faeces and stagnant water were collected for the same reason. A third mare (Horse 10) was found depressed, reluctant to move and with a rectal temperature of 40.6 °C and was transported to a stable for treatment. This mare was euthanised on 30 November after developing dyspnoea due to severe swelling of the neck and head, especially around the nares, upper lip and between the mandibular rami. A postmortem examination was carried out at the Institute for Experimental Pathology at Keldur, starting within 30 min of euthanasia.

**Fig. 1:**
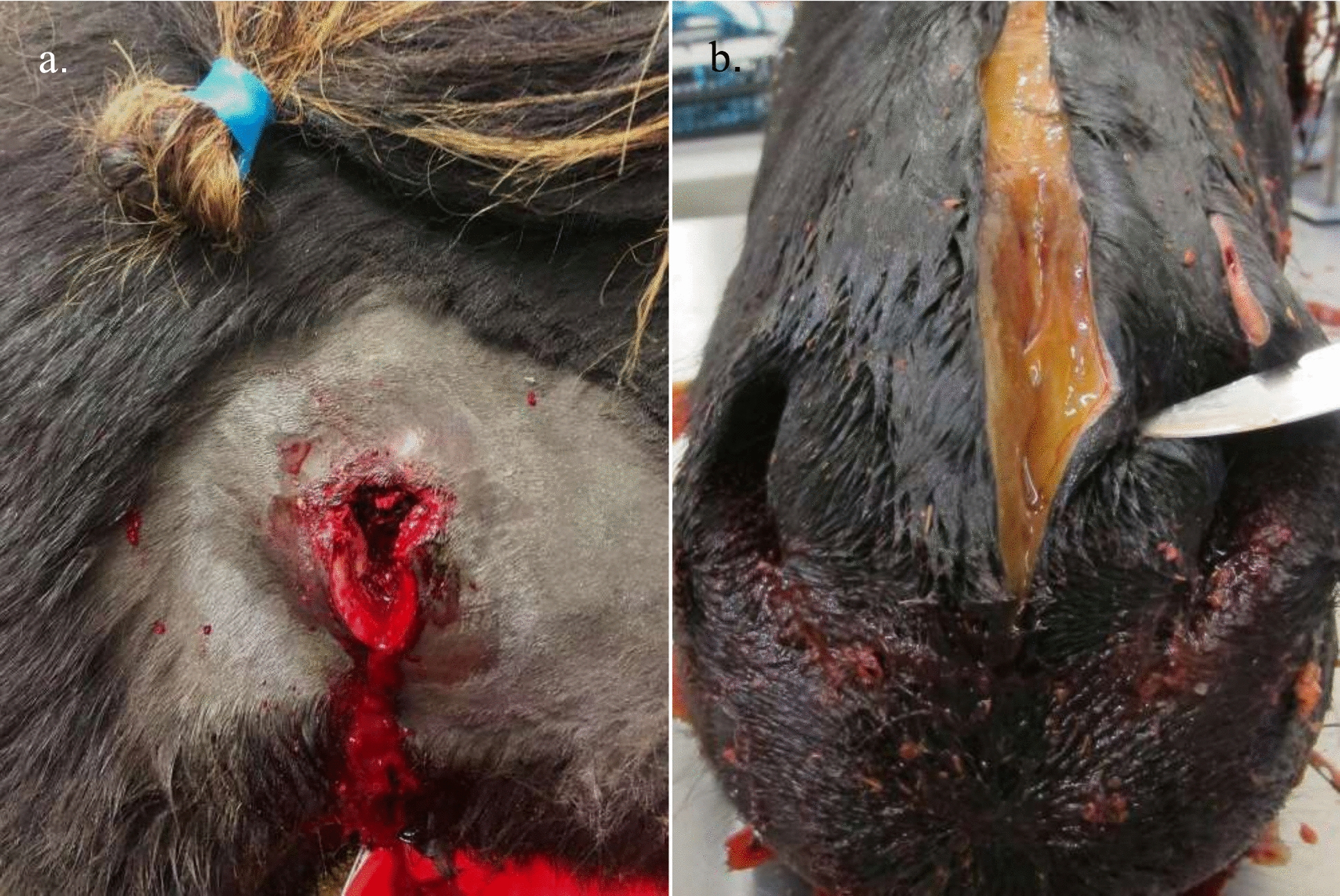
Subcutaneous changes due to *Clostridium septicum* infection in Icelandic horses. **a** Erupted subcutaneous abscess on the neck of a four-year-old mare (Horse 3) that had been injected with Noromectin ^®^ 1% solution 15 days previously. An incision was made ventral to the eruption of the abscess to drain and clean the lesion. **b** Postmortem incision into the muzzle of a six-year-old mare (Horse 10) that was euthanised due to severe clinical signs, including swelling of the head and neck. Cut surface of the swollen muzzle above and between the nares, showing severe, gelatinous, subcutaneous oedema

On 5 December, a large ulcer had formed on the chest of a seven-year-old gelding (Horse 2) and was oozing a watery, blood-tinged exudate. Four horses (Horses 4, 13, 14 and 15) had firm swollen areas of varying size on the right side of their necks corresponding to the area where injection had been performed. Over the following days, spontaneous drainage of a seropurulent exudate occurred from swellings on four more horses (Horses 3, 5, 16 and 17; Fig. 1a) and surgical fenestration was performed on one horse (Horse 13). Samples of the exudate and incised tissue were collected for bacterial culture and histopathological examination, respectively, from Horses 3, 5 and 13.

At the end of the outbreak, 16 of the 32 horses had developed noticeable clinical signs of varying severity, including three horses that died and two that were euthanised, in addition to the one horse found dead with no previously noted clinical signs at the beginning of the outbreak.

**Fig. 2:**
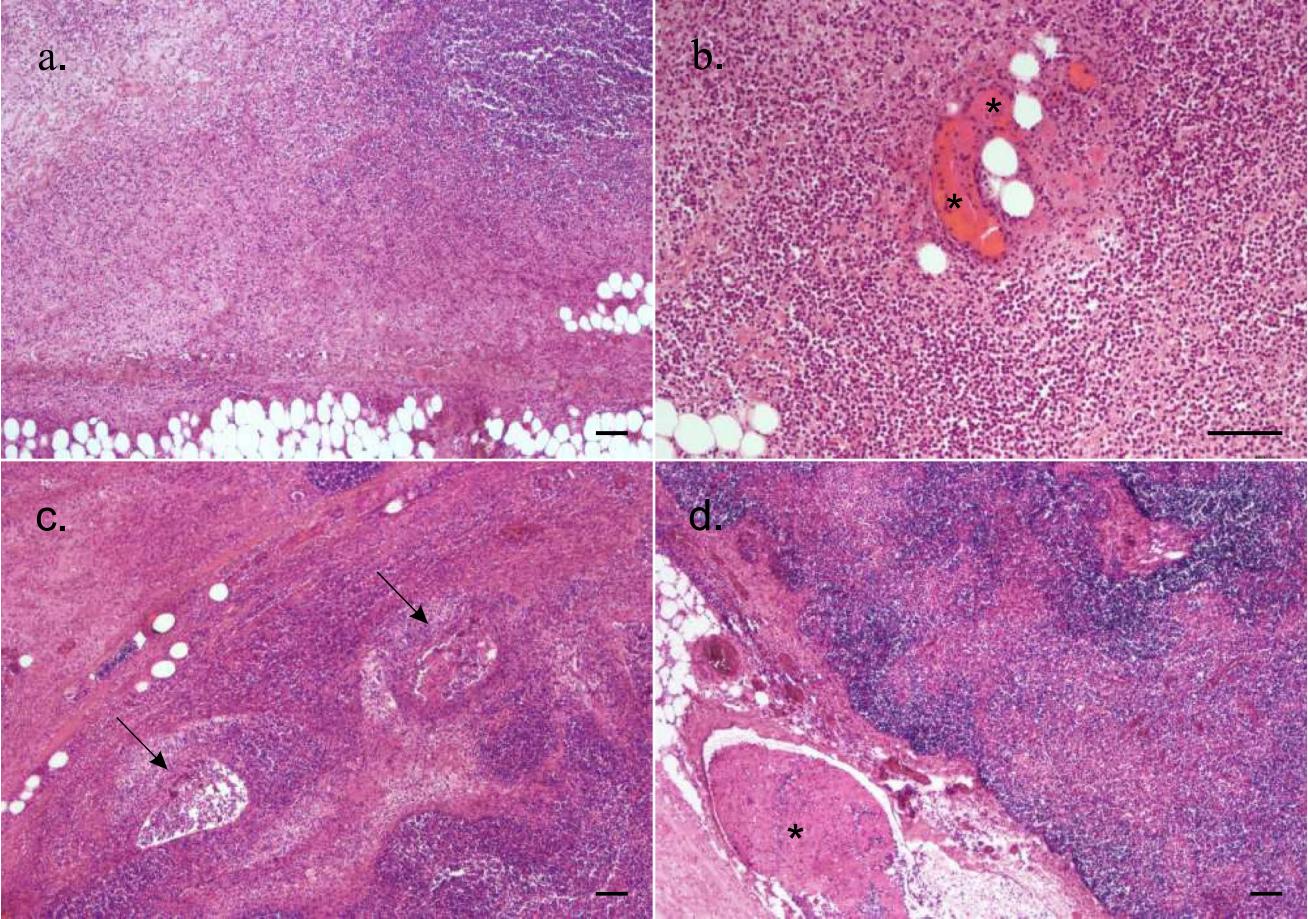
Histopathological changes in a mare with a subcutaneous *Clostridium septicum* infection. Haematoxylin–eosin-stained histological sections of lesions seen in the six-year-old mare (Horse 10) in Fig. 1b. **a** Subcutaneous tissue from the neck with diffuse inflammation and oedema of the adipose tissue. **b** Higher magnification of the subcutaneous tissue from the neck with purulent inflammation, vasculitis and vascular thrombosis (*). **c** Severely inflamed mandibular lymph node, bottom right-hand corner. The inflammation extends into the surrounding connective tissue, effacing the boundaries of the lymph node. Two lymphatic vessels are surrounded and distended by inflammatory exudate (arrows). **d** Inflamed retropharyngeal lymph node with a fibrinocellular clot in a distended lymphatic vessel in the perinodal connective tissue (*). Bar = 100 μm

Necropsy of the six-year-old mare (Horse 10), euthanised on 30 November, revealed extensive swelling of the head, especially the muzzle, and throughout the length of the ventral and lateral neck. The swelling on the muzzle was primarily due to an extensive subcutaneous, gelatinous oedema (Fig. 1b). On the neck, however, there was a uniformly firm swelling of the subcutaneous tissue that had a tan, mottled discolouration and a cloudy, pale orange fluid oozing from cut surfaces. Mild subcutaneous oedema was found on the chest and in the groin area on the left-hand side. When an axillary cut was made and the right front leg flexed laterally, a well demarcated, cylindrical, caseous abscess of approximately 20 cm appeared on the external surface of the thorax at the expected location of the axillary lymphocentre, but remnants of lymph node tissue were not seen on cut surfaces of the abscess. There was congestion of the nasal mucosa, and mild hyperaemia and petechiae in the oral, laryngeal and tracheal mucosa. Submucosal oedema was seen in the larynx and was particularly pronounced in the trachea. Lymph nodes were not noticeably enlarged, and the guttural pouches, skeletal muscles and internal organs were without specific lesions. Histological examination of samples taken from the swollen upper lip primarily showed a severe oedema in the subcutaneous tissue, with lymphatic vessels distended by neutrophils. Severe oedema, sparse haemorrhage, vasculitis, thrombosis and extensive fibrinopurulent inflammation were observed in the subcutaneous tissue originating from the swollen neck (Fig. 2a and b). There was inflammation in the medial retropharyngeal and mandibular lymph nodes that extended into the surrounding connective tissue. The perinodal tissue of the retropharyngeal lymph nodes was also oedematous and haemorrhagic. The inflammation was purulent to fibrinopurulent and involved perinodal lymphatic vessels (Fig. 2c and d). Histological sections of the caseous abscess found in the right axillary region showed interlobular to diffuse fibroplasia and neovascularisation, with pockets of fibrinous and purulent exudate in the adipose tissue peripherally to the necrotic debris of the abscess. No lymphoid tissue was present in the examined sections. No bacteria were seen in tissue sections stained with haematoxylin and eosin or Gram stain.

Virological investigations included testing peripheral blood mononuclear cells, nasal swabs and faecal samples in a pan-coronavirus reverse transcription-polymerase chain reaction (RT-PCR) [8], equine coronavirus M and N gene quantitative polymerase chain reaction (qPCR) [9], pan-influenza virus qPCR [10] and equine viral arteritis PCR. Paired serum samples from seven horses were also tested for virus-specific antibodies to equine herpesvirus type 1, equine arteritis virus, equine infectious anaemia virus and equine influenza virus H3N8. All virological and serological analyses were negative.

Examination for bacterial pathogens included non-selective clinical veterinary bacteriology [11] using sheep blood agar as basic nutritive media and SSI agar as selective media, selective *Salmonella* culture and *Listeria* culture, according to published international standards (Iso 9001, NMKL 71, 136 and 187). At the beginning of the outbreak, faecal samples and water samples from the pasture were taken for *Salmonella* culture, *Listeria* culture and diagnostic veterinary bacteriology. These samples were negative for *Salmonella* spp., *Listeria* spp. and other pathogenic bacteria.

Additional culturing for clostridia was performed on the water samples by filtering and selective enrichment. Diagnostic veterinary bacteriology was performed under aerobic and anaerobic conditions at 37 °C on organ samples and swabs of subcutaneous fluid from the necropsied horse, swabs of the exudate from the neck abscesses of three other horses, as well as neck skin scrapings and skin swabs from those horses. The same procedure was performed on the liver from the horse subjected to postmortem examination in the field. Samples from the necropsied horse were taken 45–90 min after euthanasia. The empty vial, as well as syringes and needles used in the affected herd were washed out with cooked meat broth, collected and centrifuged. The resulting sediment was cultured on blood agar under aerobic and anaerobic conditions. Diagnostic bacteriological culture of swabs from the lung, abscess, trachea, larynx and guttural pouch swabs from the necropsied horse, the purulent material from subcutaneous abscesses on three horses and the treated water samples resulted in almost pure growth of *C. septicum,* which was confirmed by traditional methods and Matrix-assisted laser desorption/ionization time-of-flight mass spectrometry, MALDI-TOF MS (Microflex LT, Bruker Daltonics, Bremen, Germany). Liver tissue from the horse examined in the field, as well as lung, liver and spleen tissue samples from the necropsied horse all yielded negative results. Skin swabs and scrapings from the neck of surviving horses with injection site abscesses revealed a sparse culture of mixed cutaneous microflora and no *C. septicum*. The vials, syringes and needles used to inject the horses yielded no specific bacterial results.

Genomic DNA for whole genome sequencing was isolated from *C. septicum* isolates cultured from a lung swab of the necropsied horse (Horse 10), a swab from a subcutaneous abscess from a surviving horse (Horse 3) and from a sample taken from stagnant water in the pasture grazed by the herd. A detailed description of the materials and methods used for the sequencing and downstream bioinformatics analyses used to derive the results is given in Additional file 1. *Clostridium septicum* assemblies and raw whole genome data available in public databanks were used for comparison to the Icelandic isolates (DRR016039, DSM 7534 (reference genome), MGYG-HGUT-02373, RMA 8861, VAT12 and WW106; details in Additional file 1). A detailed comparative analysis of this material has been published [12].

Of the genomes analysed, the shared genome fraction of the Icelandic isolates and the reference genome was 90.6% for 4105-S-STR and 4049–2, and 89.7% for the water sample isolate; their total genome length was 3,174,101 bp., 3,174,947 bp. and 3,419,405 bp., respectively (Additional file 2). Therefore, 10% of the reference genome was not present in these isolates and they differed in size, with the water isolate genome closer to the size of DSM 7534 (3,399,422 bp.). The data suggested that the *C. septicum* isolates from the horses were the same strain, while the water isolate was a separate strain (Additional files 3 and 4). Overall, the genome fraction data for all the genomes compared to the reference genome were indicative of a heterogeneous genome size within the *C. septicum* strains.

Pangenome analysis showed a core genome of 2,537 genes common to the nine *C. septicum* strains examined (Additional file 4).

The sequences of the alpha-toxin genes of all strains were compared on the amino acid and nucleotide level (Additional files 5 and 6, respectively). The amino acid sequences of the alpha-toxin protein were identical in all strains except that of the pasture water strain, which differed in four amino acids. The nucleic acid sequences differed in eight nucleic acids, of which six differences were only present in the pasture water strain sequence. This suggested that the pasture water strain was somewhat of an outlier within the strains examined.

**Fig. 3:**
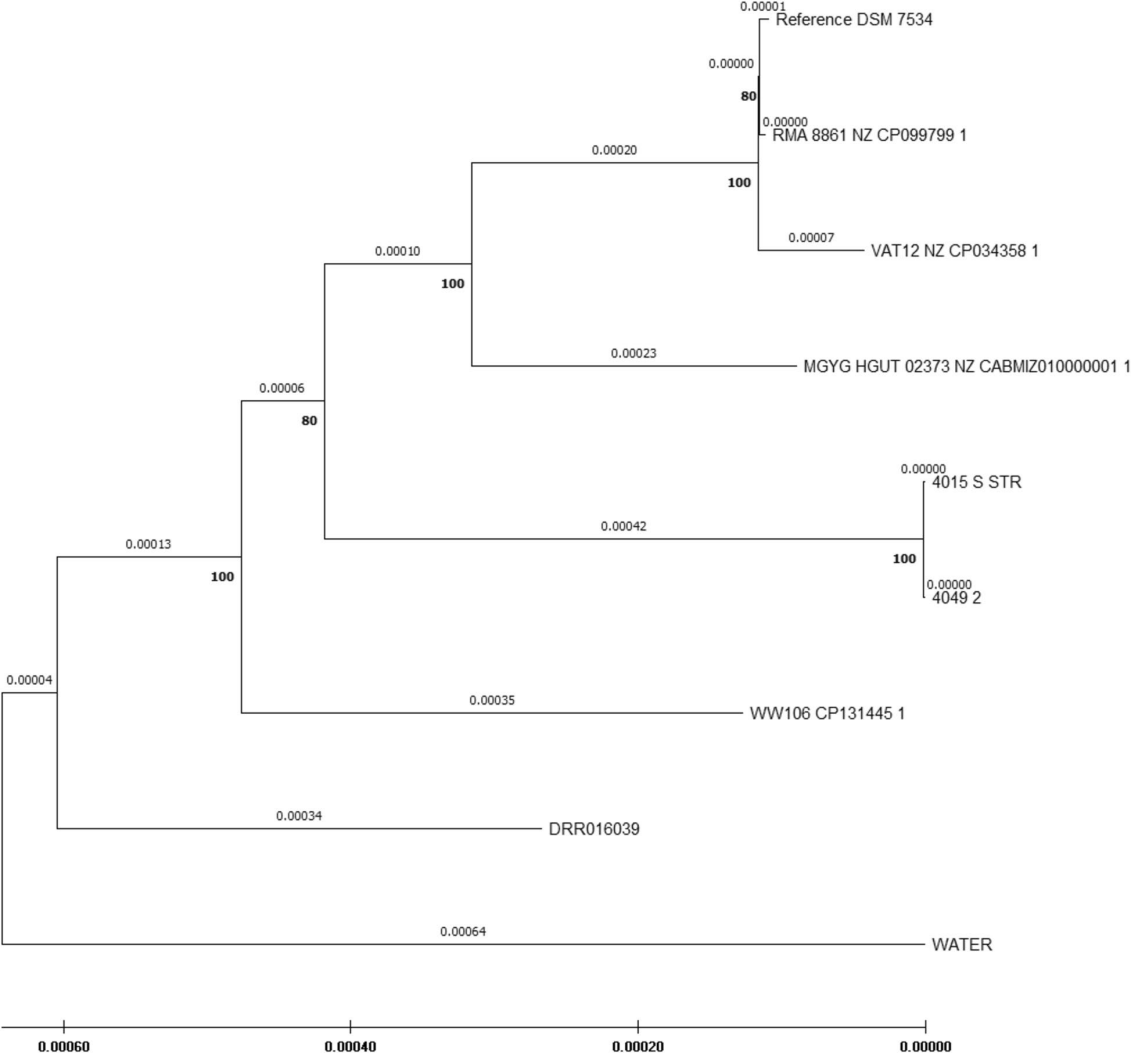
The evolutionary relationship between the genomes of the examined *Clostridium septicum* strains. A phylogenetic tree constructed from sequence alignment of the *C. septicum* strain genomes, as described in Additional file 1, including three strains originating from the outbreak described here. Strain 4049 2 originates from a lung swab of Horse 10, who was euthanised and subjected to postmortem examination due to dyspnoea and extreme swelling of the neck and head nine days after subcutaneous injection in the neck. Strain 4015 S STR originates from a subcutaneous abscess on the neck of Horse 3, which erupted approximately two weeks after subcutaneous injection in the region. Strain WATER originates from stagnant water taken from the pasture where the horses were kept. The figure shows branch lengths in substitutions per site. The numbers by the nodes show bootstrap values

Sequence reads (Icelandic strains and DRR016039) and assembled contigs (VAT12, RMA 8861, MGYG-HGUT-02373 and WW106) were mapped to the reference sequence (DSM 7534) to generate an alignment. The alignment was analysed for putative recombinant regions. The alignment, excluding putative recombinant regions, was used to construct a phylogenetic tree using maximum likelihood estimation (Fig. 3). Based on the alpha-toxin alignments, the pasture water strain was designated as an “outlier” in the tree.

## Discussion and conclusions

For the case described here, we hypothesise that a herd of horses developed severe subcutaneous infection with *C. septicum* after receiving an injection with an ivermectin formulation through contaminated skin. Infection with *C. septicum* in horses, typically in the form of gas gangrene, has been reported in other parts of the world, especially the Americas [5, 6, 13], but never in Iceland, and it is therefore extremely important to highlight this risk for Icelandic equine clinicians in this country.

Consequently, gas gangrene was not the initial focus of the investigation, although on the list of differential diagnoses. The collection of samples and diagnostic approach was therefore not aimed at optimising the isolation of clostridia. The case description is therefore less focused than would be ideal, but the authors feel that it is important to report on the risk of causing clostridial infection by injecting through contaminated skin in horses, as the practice of subcutaneous anthelmintic injection is widespread in Iceland.

The necropsy of one horse 9 days after injection revealed lesions consistent with malignant oedema, characterised by thick, gelatinous subcutaneous oedema and tan discolouration of underlying muscles with involvement of lymph nodes in surrounding connective tissue. Swabs of subcutaneous fluid from the affected region of the head and neck, and the respiratory tract, taken 45–90 min after euthanasia of the affected horse yielded almost pure growth of *C. septicum*, whereas tissue samples of lung, spleen and liver yielded no bacterial growth. As the samples were taken shortly after euthanasia and in a necropsy facility, postmortem contamination of the samples by *C. septicum* seems unlikely.

Injection of Noromectin® was common to all horses involved in the outbreak, and lesions in some of the surviving horses were found around the injection site. The horses were kept in an area of peatland with wet soil rich in organic material, which can contain more clostridium spores than drier, more mineral soil [13]. The coat and skin of the horses could therefore have been contaminated from rolling in this soil, although when bacterial culture was attempted on the surviving horses over 2 weeks later, no *C. septicum* was cultured.

Previous fatal equine disease outbreaks in Iceland have been caused by *Salmonella* infection [14], ingestion of haylage contaminated by *C. botulinum* neurotoxin [1, 2] or *Listeria monocytogenes* [15, 16]. The pasture where the affected horses in the present outbreak were kept is in a region with previous outbreaks of salmonellosis in horses, often in relation to stagnant water. As the initial clinical signs (including high fever) were nonspecific, the first response of veterinary investigators was to focus on epidemiological information and sample collection relating to salmonellosis, listeriosis and the potential involvement of equine influenza virus, equine arteritis virus or other viruses never identified in Iceland. Botulism was quickly excluded as the horses had not been fed haylage and clinical signs were not indicative for this condition. Soon after most of the fatalities had occurred, the development of clinical signs among survivors and diagnostic results from the abovementioned samples reduced the likelihood that any of these bacterial and viral agents were the cause of the disease. The varying clinical manifestations in this outbreak illustrate that the outcome of an infection with *C. septicum* is likely to rely on many factors [17]. The clinical manifestations included severe systemic disease (in some cases this was fatal soon after clinical signs were detected), protracted cellulitis and abscesses. *C. septicum* infections in horses have been known to cause death within hours of the onset of clinical signs and only 3 days after intramuscular injection [18]. As a horse found dead with no previously noted clinical signs was the first fatality in the present outbreak, no examination or sample collection was carried out on that carcass.

It is important to note that the number of horses affected by the infection may have been higher than recorded by the owners, as they may not have noticed clinical signs in all horses kept on pasture. As previously described, crepitus due to subcutaneous gas accumulation is an inconsistent finding in clostridial cellulitis [19]. Other, more visible signs associated with *C. septicum* cellulitis or myositis, such as signs of abdominal pain, treading and pawing [19, 20], were not noted during this outbreak.

The cases of gas gangrene or cellulitis in horses due to *C. septicum* described in the literature tend to involve only one animal [13, 18, 19, 21]. In Iceland, horses are often kept in herds, and in many respects are treated as herd animals. In other countries, clostridial infections have been described in multiple individuals of other animal species after herd treatment, such as vaccinations [3, 22, 23]. *Clostridium sordellii* was found to cause clinical signs and the death of 63 out of 1,000 sheep in Brazil that were vaccinated using the same needle for the whole flock [3]. The authors believed this indicated that the contaminant organism was present in the environment and on the skin of the animals [3]. In the outbreak described here, one and the same needle and syringe were used for four to five horses consecutively before taking a new set for the next four to five animals, and injections were administered without clipping the coat or disinfecting the skin. This has been common practice in Iceland for more than three decades. As outbreaks of this magnitude have not previously been reported, it can be speculated that the skin of the affected horses was heavily contaminated as was presumed in the reported outbreak in sheep [3]. In the current case, the first horse to be injected was a mare that died 6 days post-injection, after having a fever and showing reluctance to move for at least 2 days, indicating that even with a sterile needle, enough spores might have existed on the skin to cause serious disease when introduced by puncturing the skin. The risk seems to have been higher for the horses kept on the larger pasture or that came in close contact with horses kept there, indicating soil contamination with *C. septicum*. Furthermore, epidemiological data indicate that the onset and nature of the clinical signs depended on the infective dose. Merely three days post-injection, pyrexia and stiff movement were noted in three horses (Horses 1–3, Table 1) whose skin must have been heavily contaminated. In addition, these three horses most likely transferred this contamination on to the skin of Horse 4 that had never been on pasture with the others but was transported by trailer with Horses 1–3, stabled with them and injected 2 days later. This horse showed pyrexia and oedema 2 days after injection. No soil samples were collected in this investigation, only pasture water samples, in line with the initial suspicion of a waterborne *Salmonella* infection. Although *C. septicum* was isolated from the water samples, the strain was a distant relative of the disease-causing strain found in the horses. However, the presence of this isolate indicates that *C. septicum* was prevalent in the horses’ environment.

In the outbreak described here, only one horse was subjected to necropsy, and it is therefore only possible to describe the pathological changes seen in this individual. No clear focus of tissue reaction consistent with the subcutaneous injection of ivermectin was observed, but as euthanasia was performed 9 days after injection, the original focus might have been lost due to dissemination of the subcutaneous tissue reaction. In Argentina, a postmortem examination of a sheep that died 24 h after blood sampling revealed subcutaneous oedema around three pin-point haemorrhagic lesions left by venepuncture, from which *C. septicum* and *C. sordellii* were cultured [24]. In the present outbreak, this clear association between lesions and the site of puncture might have been masked due to prolonged pathological changes in the affected region. The caseous abscess found in the right axillary region of the necropsied horse was initially thought to be inconsistent with clostridial infection, but the location and encapsulated nature of the abscess indicated that this was the axillary lymphocentre, which drains the skin of the caudal neck and shoulder, the region of the injection site. Therefore, even if clostridia were introduced subcutaneously, they could have been brought to the axillary lymphocentre by lymphatic drainage and gravitational force in the 9 days that followed. Abscess formation in horses has been reported to be a consequence of infection with *C. septicum*, albeit rarely [25], *C. perfringens* [18, 26] and *Clostridium novyi* [27], and necrosuppurative infection with *C. sordellii* has also been described [6]. Swabs from the guttural pouch, larynx, trachea and lung revealed *C. septicum*, but no bacteria were cultured from deep in the lung tissue itself, nor liver or spleen. This indicates that during the protracted disease process with extremely difficult breathing, the bacteria spread to nearby structures of the pharynx and respiratory tract, both with breathing and via the lymph vessels.

The results of the whole genome sequencing analysis showed that the *C. septicum* strain from the necropsied horse and the strain from the subcutaneous abscess from the surviving horse were the same. In contrast, the *C. septicum* strain from the pasture water sample differed from both this strain and the *C. septicum* strains from other geographical locations, available in public databanks. The sequencing was performed to give an epidemiological perspective on the Icelandic strains in terms of publicly available *C. septicum* genome data. However, the paucity of genome data for *C. septicum* in public databases hampered conclusions on the origin of the strain that caused disease in the horses. The data suggest that the strain isolated from the pasture water sample was relatively evolutionarily distant to the other genomes analysed, including data obtained from public databanks. It is of interest that the water strain was the only one where amino acid variability was detected in the alpha-toxin sequence. Data shown in Fig. 3 underline the heterogeneity of the available *C. septicum* genomes. Our results show the necessity for sequence data on more *C. septicum* strains from diverse locations for a better understanding of the variability within the species and within Iceland. One way to approach this within Iceland would be by systematic WGS analysis of both pathogenic and environmental strains within the country.

It was speculated whether spores were present in the drug product, but this was not supported by epidemiological data. The remainder of a vial with the same batch number used in this herd was subsequently used in other horses in a different location without causing any clinical signs. Furthermore, no clostridia were cultured from the empty vial, syringes and needles used in the affected herd. Contamination of the puncture wound with clostridial spores from the environment appears to have led to the growth of *C. septicum* and establishment of an active infection. Although the injected ivermectin product is unlikely the source of clostridial spores, the accompanying leaflet for Norodine^®^, revised in September 2020, describes discomfort and tissue swelling in injected cattle, associated with clostridial infection. As with other injectable ivermectin products, Norodine^®^ is not marketed for horses due to results of the original avermectin development programme, where 2.4 injection site infections and 1.5 deaths were reported per 100,000 horse doses sold. Therefore the parenteral formulation originally developed by Merck was withdrawn after 17 months on the U.S. market [28]. Exceptionally heavy clostridial contamination of the environment and skin has been found to be a risk factor in sheep [3, 22]. In studies describing clostridial infection of the subcutis or muscles, it is likely that local tissue damage caused by injecting a tissue debilitant leads to favourable conditions for clostridia to multiply and establish an active infection [17, 18, 20].

In conclusion, the injection of 32 horses with an anthelmintic product predisposed them to serious subcutaneous infection by *C. septicum*. Over half of the horses developed clinical signs, of which one-third died or were euthanised. No similar outbreaks have previously been reported in Iceland, although subcutaneous anthelmintic formulations have been widely used for more than 30 years. The outbreak described in this report warrants a reassessment of this practice due to the reported risk of *C. septicum* contamination in the environment of horses in Iceland. As the source of contamination in the affected horses’ environment was not identified, this risk should be considered more widely throughout the country. More information on the distribution and variability of *C. septicum* strains is clearly needed to give a better understanding of the risk posed to horses.

## Supplementary Information

Additional file 1. A detailed description of the materials and methods used for the sequencing and downstream bioinformatics analyses.

Additional file 2. Results from a QUAST analysis of genome assemblies.

Additional file 3. Results from genome annotation using prokka on the assemblies of the Icelandic strains, DRR016039, and the NCBI assemblies.

Additional file 4. Results from roary analyses on the assemblies of the Icelandic strains, DRR016039, and the NCBI assemblies.

Additional file 5. Results from multiple sequence alignment of the amino acid and nucleotide sequences of the alpha-toxin genes in all *Clostridium septicum* strains included in the study.

Additional file 6. A summary of the results from the ClonalFrameML analysis of recombination in the genome assemblies for all of the *Clostridium septicum* strains.

## Data Availability

The raw whole genome sequencing data for the three Icelandic strains was deposited in the European Nucleotide Archive (https://www.ebi.ac.uk/ena/browser/home) under the project/accession number PRJEB70871.

## Declarations

### Prior publication

Data have not been published previously.
