# Correction: Severe subcutaneous infection with *Clostridium septicum* in a herd of native Icelandic horses

**DOI:** 10.1186/s13028-025-00799-5

**Published:** 2025-03-17

**Authors:** Charlotta Oddsdóttir, Ólöf G. Sigurðardóttir, Vala Friðriksdóttir, Vilhjálmur Svansson, Birkir Þór Bragason, Sigríður Björnsdóttir

**Affiliations:** 1 Division of Bacteriology and Pathology, Institute for Experimental Pathology at Keldur, Keldnavegi 3, 112 Reykjavík, Iceland; 2 Division of Virology, Molecular Biology and Parasitology, Institute for Experimental Pathology at Keldur, Keldnavegi 3, 112 Reykjavík, Iceland; 3 Icelandic Food and Veterinary Authority, Austurvegi 64, 800 Selfoss, Iceland

**Correction: Acta Veterinaria Scandinavica (2025) 67:8**

10.1186/s13028-025-00792-y

Following publication of the original article [1], we have been notified that there were unnecessary lines published in Fig. 2. Also, Table 1 was published incorrectly.

