# Supplementary material for: Severe subcutaneous infection with Clostridium septicum in a herd of native Icelandic horses
Source: Acta Vet Scand. 2025 Feb 6;67:8. doi: 10.1186/s13028-025-00792-y (PMC11800538; doi:10.1186/s13028-025-00792-y)
Supplement: Supplementary file 1 — Additional file 1. A detailed description of the materials and methods used for the sequencing and downstream bioinformatics analyses. [file 13028_2025_792_MOESM1_ESM.pdf]

## Materials and methods of genome analyses

### DNA isolation and whole genome sequencing

DNA isolation was performed using a GeneJet Genomic DNA Purification Kit (Thermo Scientific, Catalog #K0721). The isolated genomic DNA was sent to BGI Genomics for library construction (Low input DNA library-Vazyme) and whole genome sequencing (WGS) utilizing the DNBSEQ platform with paired-end reads of 150 bp in length. The statistics of the clean WGS data are shown below:

**Table 1. Statistics of clean sequencing data for the Icelandic isolates**

| Sample name | Clean reads | Clean base | Read length | Q20(%) | Q30(%) | GC(%) |
|---|---|---|---|---|---|---|
| 4015_S_STR | 4,066,586 | 1,219,975,800 | PE150 | 97.86 | 94.29 | 28.96 |
| 4049_2 | 4,044,671 | 1,213,401,300 | PE150 | 97.91 | 94.43 | 28.62 |
| Water | 4,101,618 | 1,230,485,400 | PE150 | 98 | 94.66 | 29.34 |

### Taxonomic classification of Icelandic isolates and publically available sequence data used in the study.

Correct taxonomic classification of the WGS reads was done using Kraken 2 (1) and Bracken (2). The sequence of the reference *C. septicum* type strain, DSM 7534 (NCBI reference sequence NZ_CP023671.1), was obtained from the NCBI website. A search for *C. septicum* raw read genome data was performed on the NCBI database (NCBI: National Center for Biotechnology Information) and that of the European Nucleotide Archive (ENA). Raw data of sufficient quality from Illumina sequencing of one *C. septicum* isolate was found in the ENA database, accession number DRR016039 (origin unknown). The raw sequence quality of the Icelandic isolates and DRR016039 was evaluated using fastqc v.0.11.9 (Babraham Bioinformatics). Trimmomatic (3) was used for processing of the raw read data based on the fastqc quality assessment. Fully assembled, unique, *C. septicum* genomes were obtained from the NCBI database: VAT12 (accession (acc.) nr. NZ_CP034358, assembled genome: 3.454.144 bp., strain isolated from wild Turkey (*Meleagris gallopova*), USA, Virginia, Shenandoah Valley, 2012-02-13), RMA 8861 (acc. nr. NZ_CP099799, assembled chr.: 3.375.247 and plasmid: 5296), MGYG-HGUT-02373 (acc. nr. NZ_CABMIZ000000000, assembled contigs, strain isolated from human gut), and

WW106 (acc. nr. CP131445, assembled genome: 3.439.412, strain isolated from wastewater of an animal research facility).

### *De novo* assembly

*De novo* assembly of the raw sequence data of the three Icelandic isolates and DRR016039 was performed with SKESA (4) using default parameters. The assemblies were corrected using Pilon (5). Quality assessment of the assemblies was performed with QUAST (6) (Additional file 2). with reference to genome data for the reference genome DSM 7534.

### Genome annotation and pangenome analysis

The corrected genome assemblies and the databank assemblies were annotated with Prokka (7) using protein annotation data available for the reference genome (DSM 7534, NCBI) (Additional file 3). The prokka output was then used for pangenome analysis with Roary (8) (Additional file 4).

### Analysis of the alpha-toxin gene

The assemblies for 4105-S-STR, 4049-2, DRR016039, and the water strain were screened for antimicrobial resistance genes and virulence genes using ABRicate (https://github.com/tseemann/abricate) which compares input to data in the following databases NCBI AMRFinderPlus, CARD, Resfinder, ARG-ANNOT, VFDB, PlasmidFinder, EcOH, and MEGARES 2.00. All databases were updated on the date of analyses (2023-Oct-30). The only hit was obtained with the VFDB database and consisted of the alpha-toxin gene (accession number ACA60977) in all strains. The sequence (1332 bp.) of the alpha-toxin gene of all nine strains examined in this study were compared on the nucleotide and protein level using Clustal-Omega (9) to generate multiple sequence alignments (Additional file 5).

### Phylogenetic analysis

Sequence reads from the Icelandic strains and DRR016039 in addition to the assembled contigs of VAT12, RMA 8861, MGYG-HGUT-02373, and WW106, were mapped to the reference sequence (DSM 7534) using snippy v4.6.0 (https://github.com/tseemann/snippy). The core alignment from snippy was used to generate a tree using raxmlGUI 2.0 (10). The tree and

alignment were used as input into ClonalFrameML (11)to identify recombination. The ClonalFrameML analysis revealed recombinant regions in the strains analysed in this study (Additional file 6). Recombinant regions were masked in the original snippy alignment using maskrc-svg (https://github.com/kwongj/maskrc-svg#maskrc-svg). The masked alignment was used to construct a phylogenetic tree for the *C. septicum* strains with raxmlGUI 2.0 (Figure X). Based on the alpha-toxin data, the pasture water strain was designated as an "outlier" in the tree.

## References


1. Wood DE, Lu J, Langmead B. Improved metagenomic analysis with Kraken 2. Genome Biol. 2019;20(1):257.
2. Lu J, Rincon N, Wood DE, Breitwieser FP, Pockrandt C, Langmead B, et al. Metagenome analysis using the Kraken software suite. Nat Protoc. 2022;17(12):2815-39.
3. Bolger AM, Lohse M, Usadel B. Trimmomatic: a flexible trimmer for Illumina sequence data. Bioinformatics. 2014;30(15):2114-20.
4. Souvorov A, Agarwala R, Lipman DJ. SKESA: strategic k-mer extension for scrupulous assemblies. Genome Biol. 2018;19(1):153.
5. Walker BJ, Abeel T, Shea T, Priest M, Abouelliel A, Sakthikumar S, et al. Pilon: an integrated tool for comprehensive microbial variant detection and genome assembly improvement. PLoS One. 2014;9(11):e112963.
6. Mikheenko A, Prjibelski A, Saveliev V, Antipov D, Gurevich A. Versatile genome assembly evaluation with QUAST-LG. Bioinformatics. 2018;34(13):i142-i50.
7. Seemann T. Prokka: rapid prokaryotic genome annotation. Bioinformatics. 2014;30(14):2068-9.
8. Page AJ, Cummins CA, Hunt M, Wong VK, Reuter S, Holden MT, et al. Roary: rapid large-scale prokaryote pan genome analysis. Bioinformatics. 2015;31(22):3691-3.
9. Madeira F, Pearce M, Tivey ARN, Basutkar P, Lee J, Edbali O, et al. Search and sequence analysis tools services from EMBL-EBI in 2022. Nucleic Acids Res. 2022;50(W1):W276-W9.
10. Edler D, Klein J, Antonelli A, Silvestro D. raxmlGUI 2.0: A graphical interface and toolkit for phylogenetic analyses using RAxML. Methods Ecol Evol. 2021;12(2):373-7.

11. Didelot X, Wilson DJ. ClonalFrameML: Efficient Inference of Recombination in Whole Bacterial Genomes. Plos Comput Biol. 2015;11(2).
