# Supplementary material for: Severe subcutaneous infection with Clostridium septicum in a herd of native Icelandic horses
Source: Acta Vet Scand. 2025 Feb 6;67:8. doi: 10.1186/s13028-025-00792-y (PMC11800538; doi:10.1186/s13028-025-00792-y)
Supplement: Supplementary file 2 — Additional file 2. Results from a QUAST analysis of genome assemblies. [file 13028_2025_792_MOESM2_ESM.pdf]

Additional file 2. Results from QUAST analysis of assemblies. QUAST (Quality Assessment Tool for Genome Assemblies) (1, 2) is a tool to determine the quality of genomes assemblies by certain metrics shown in the table below. The top two parts of the table, i.e. "Genome statistics" and "Misassembles" show data relative to the reference genome DSM 7534. The lowest part of the table "Statistics without reference" shows assembly data that is independent of the reference genome. In this study assembly was performed with SKESA for the Icelandic isolates and DRR016039, whereas the assembled data for WW106, VAT12, RMA_8861, and MGYG-HGUT-02373 was obtained from the NCBI database.

NGA50: Modified N50 statistic: 50 % of the reference genome in question, in this case DSM 7534, was in contigs equal to, or longer, than the NGA50 number. As shown in the table, the NGA50 for the assemblies of 4015_S_STR, 4049_2, Water, and DRR016039 was 27336 bp., 27336 bp., 30028 bp., and 31501 bp., respectively.

Genome fraction (%): This statistic shows the percentage of shared sequence between the strain in question and the reference genome DSM 7534. As shown in the table, the aligned genome fraction was variable for the *C. septicum* strains relative to the reference genome. The shared percentage of the reference genome and that of 4015_S_STR, 4049_2 and the water isolate was 90.591 %, 90.621 %, and 89.731 %, respectively.

Total length: This statistic shows the cumulative length of the assembled contigs for the Icelandic isolates and DRR016039 which is an indicator of genome size. The total lengths of assembled contigs for 4015_S_STR, 4049_2, and the water isolate, were 3.174.101 bp., 3.174.947 bp., and 3.419.405 bp., respectively.

**Genome statistics**	**4015_S_STR**	**4049_2**	**WW106**	**DRR016039**	**VAT12**	**RMA_8861**	**MGYG-HGUT-02373**	**Water**
Genome fraction (%)	90.591	90.621	96.08	90.937	93.273	99.282	91.95	89.731
Duplication ratio	1.001	1.001	1.017	1.001	1.039	1	1.004	1.002
# genomic features	2834 + 52 part	2838 + 52 part	3025 + 50 part	2832 + 59 part	2911 + 66 part	3162 + 2 part	2899 + 69 part	2806 + 57 part
Largest alignment	133944	133945	168256	110951	158091	2340302	181601	174852
Total aligned length	3085533	3086066	3323687	3098227	3295832	3380315	3136162	3058097
NGA50	27336	27336	53911	31501	38143	2340302	44689	30028
LGA50	34	34	20	33	27	1	25	33
**Misassemblies**								
# misassemblies	38	36	172	48	230	1	45	53
Misassembled contigs length	1302005	1245800	3439412	1529092	3454144	3375247	1899997	1486464
Mismatches								
# mismatches per 100 kbp	146.16	145.78	157.43	147.8	84.14	24.73	100.33	194.82
# indels per 100 kbp	10.21	10.24	11.37	9.33	38.64	4.5	7.79	13.06
# N's per 100 kbp	0	0	0	0	0	0	0.15	0
**Statistics without reference**								
# contigs	126	127	1	116	1	2	79	105
Largest contig	238073	225700	3439412	110982	3454144	3375247	213922	241314
Total length	3174101	3174947	3439412	3236013	3454144	3380543	3298970	3419405
Total length (>= 1000 bp)	3174101	3174947	3439412	3236013	3454144	3380543	3298970	3419405
Total length (>= 10000 bp)	2989468	2991920	3439412	3084415	3454144	3375247	3193457	3259293
Total length (>= 50000 bp)	1198635	1186048	3439412	1630135	3454144	3375247	2244719	2043299

References

1. Gurevich A, Saveliev V, Vyahhi N, Tesler G. QUAST: quality assessment tool for genome assemblies. Bioinformatics. 2013;29(8):1072-5.
2. Mikheenko A, Prjibelski A, Saveliev V, Antipov D, Gurevich A. Versatile genome assembly evaluation with QUAST-LG. Bioinformatics. 2018;34(13):i142-i50.
