# Supplementary material for: Severe subcutaneous infection with Clostridium septicum in a herd of native Icelandic horses
Source: Acta Vet Scand. 2025 Feb 6;67:8. doi: 10.1186/s13028-025-00792-y (PMC11800538; doi:10.1186/s13028-025-00792-y)
Supplement: Supplementary file 3 — Additional file 3. Results from genome annotation using prokka on the assemblies of the Icelandic strains, DRR016039, and the NCBI assemblies. [file 13028_2025_792_MOESM3_ESM.pdf]

Additional file 3. Results from genome annotation using prokka on the assemblies of the Icelandic strains, DRR016039, and the NCBI assemblies. The table shows genome features for each strain obtained using prokka (1) . The features listed are as follows: number of contigs in the assembly (contigs), the total number of bases for the strain genome (Bases), numer of coding DNA sequences (CDS), number of genes (gene), number of regions coding for miscellaneous types of RNA (Misc_RNA), number of ribosomal RNA sequences (rRNA), number of repeat regions (Repeat region), number of transfer RNA sequences (tRNA), and number of transfer-messenger RNA sequences (tmRNA).

	Water	MGYG-HGUT-02373	4049_2	4015_S	RMA_8861	VAT_12	DSM_7534	DRR016039	WW106
**Contigs**	131	79	151	152	2	1	2	147	1
**Bases**	3431914	3298970	3186697	3187427	3380543	3454144	3404718	3252064	3439412
**CDS**	3240	3100	2981	2982	3148	3507	3170	3082	3196
**Gene**	3372	3248	3108	3106	3323	3675	3345	3178	3373
**Misc_RNA**	59	53	55	55	55	56	55	57	56
**rRNA**	8	14	10	9	33	33	33	6	33
**Repeat_region**	3	1	1	1	3	2	3	2	1
**tRNA**	65	81	62	60	87	79	87	32	87
**tmRNA**								1	1

References

1. Seemann T. Prokka: rapid prokaryotic genome annotation. Bioinformatics. 2014;30(14):2068-9.
