# Supplementary material for: Severe subcutaneous infection with Clostridium septicum in a herd of native Icelandic horses
Source: Acta Vet Scand. 2025 Feb 6;67:8. doi: 10.1186/s13028-025-00792-y (PMC11800538; doi:10.1186/s13028-025-00792-y)
Supplement: Supplementary file 4 — Additional file 4. Results from roary analyses on the assemblies of the Icelandic strains, DRR016039, and the NCBI assemblies. [file 13028_2025_792_MOESM4_ESM.pdf]

Additional file 4. Results from pangenome analysis using roary on the assemblies of the Icelandic strains, DRR016039, and the NCBI assemblies. The figure below summarizes the results from roary (1) analysis on all *C. septicum* strains included in the genome analyese. Panel *a* shows the distribution of genes into the core genes (n=2537), shell (n=899), and cloud (n=1538). Core genes are defined as genes that are present in all strains, shell genes are defined as genes that are found in a subset of the strains but not all, whereas cloud genes are defined as genes that are present in only a few strains. Panel *b* shows the roary matrix indicating shared genes (core) and accessory genes (shell and cloud) that differ between the strains as well as a tree (to the left of the matrix) describing the relationship between the strains.

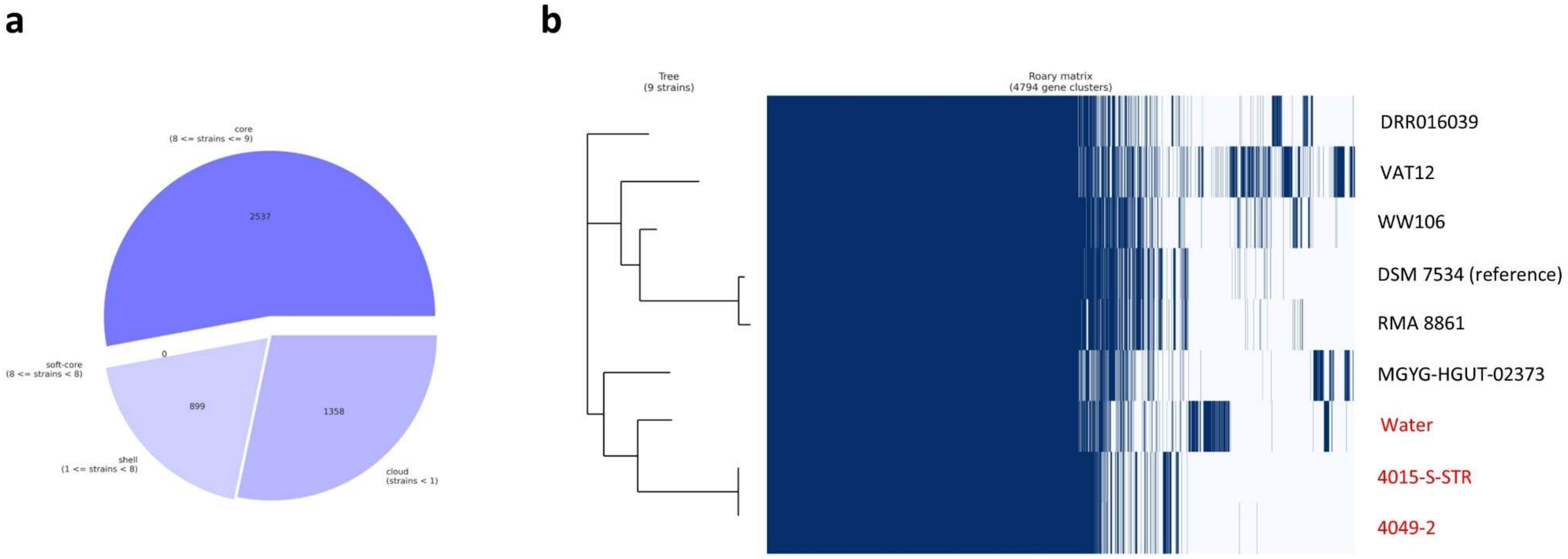

References

1. Page AJ, Cummins CA, Hunt M, Wong VK, Reuter S, Holden MT, et al. Roary: rapid large-scale prokaryote pan genome analysis. Bioinformatics. 2015;31(22):3691-3.
