# Supplementary material for: Severe subcutaneous infection with Clostridium septicum in a herd of native Icelandic horses
Source: Acta Vet Scand. 2025 Feb 6;67:8. doi: 10.1186/s13028-025-00792-y (PMC11800538; doi:10.1186/s13028-025-00792-y)
Supplement: Supplementary file 5 — Additional file 5. Results from multiple sequence alignment of the amino acid and nucleotide sequences of the alpha-toxin genes in all Clostridium septicum strains included in the study. [file 13028_2025_792_MOESM5_ESM.pdf]

Additional file 5. Result from multiple sequence alignment of the amino acid and nucleotide sequences of the alpha-toxin genes in all *C. septicum* strains included in the study (PDF). Clustal Omega (1) was used to align the amino acid sequences and nucleotide sequences of the C. septicum strains used in the study. In the amino acid alignment differences are highlighted in red. In the nucleotide alignment differences are highlighted in green if they are present in strains other than that from the water isolate or red if they are present only in the water strain.

```
CLUSTAL O(1.2.4) multiple sequence alignment


Water_sample          MSKKSFAKKVICTSMIAIQCAAVVPHVQAYALTNFEEGGYANHNNASSIKIFGYEDNEDL    60
4015_S_STR            MSKKSFAKKVICTSMIAIQCAAVVPHVQAYALTNLEEGGYANHNNASSIKIFGYEDNEDL    60
4049_2                MSKKSFAKKVICTSMIAIQCAAVVPHVQAYALTNLEEGGYANHNNASSIKIFGYEDNEDL    60
WW106                 MSKKSFAKKVICTSMIAIQCAAVVPHVQAYALTNLEEGGYANHNNASSIKIFGYEDNEDL    60
DSM_7534              MSKKSFAKKVICTSMIAIQCAAVVPHVQAYALTNLEEGGYANHNNASSIKIFGYEDNEDL    60
DRR016039             MSKKSFAKKVICTSMIAIQCAAVVPHVQAYALTNLEEGGYANHNNASSIKIFGYEDNEDL    60
VAT12                 MSKKSFAKKVICTSMIAIQCAAVVPHVQAYALTNLEEGGYANHNNASSIKIFGYEDNEDL    60
RMA8861               MSKKSFAKKVICTSMIAIQCAAVVPHVQAYALTNLEEGGYANHNNASSIKIFGYEDNEDL    60
MGYG_HGUT_02373       MSKKSFAKKVICTSMIAIQCAAVVPHVQAYALTNLEEGGYANHNNASSIKIFGYEDNEDL    60
                      **********************************:*************************

Water_sample          KAKIIQDPEFIRNWANVAHSLGFGWCGGTANPNVGQGFEFKREVGAGGKVSYLLSARYNP    120
4015_S_STR            KAKIIQDPEFIRNWANVAHSLGFGWCGGTANPNVGQGFEFKREVGAGGKVSYLLSARYNP    120
4049_2                KAKIIQDPEFIRNWANVAHSLGFGWCGGTANPNVGQGFEFKREVGAGGKVSYLLSARYNP    120
WW106                 KAKIIQDPEFIRNWANVAHSLGFGWCGGTANPNVGQGFEFKREVGAGGKVSYLLSARYNP    120
DSM_7534              KAKIIQDPEFIRNWANVAHSLGFGWCGGTANPNVGQGFEFKREVGAGGKVSYLLSARYNP    120
DRR016039             KAKIIQDPEFIRNWANVAHSLGFGWCGGTANPNVGQGFEFKREVGAGGKVSYLLSARYNP    120
VAT12                 KAKIIQDPEFIRNWANVAHSLGFGWCGGTANPNVGQGFEFKREVGAGGKVSYLLSARYNP    120
RMA8861               KAKIIQDPEFIRNWANVAHSLGFGWCGGTANPNVGQGFEFKREVGAGGKVSYLLSARYNP    120
MGYG_HGUT_02373       KAKIIQDPEFIRNWANVAHSLGFGWCGGTANPNVGQGFEFKREVGAGGKVSYLLSARYNP    120
                      ************************************************************

Water_sample          NDPYASGYRAKDRLSMRISNVRFVIDNDSIKLGTPKVKKLAPLNSASFDLINESKTESKL    180
4015_S_STR            NDPYASGYRAKDRLSMKISNVRFVIDNDSIKLGTPKVKKLAPLNSASFDLINESKTESKL    180
4049_2                NDPYASGYRAKDRLSMKISNVRFVIDNDSIKLGTPKVKKLAPLNSASFDLINESKTESKL    180
WW106                 NDPYASGYRAKDRLSMKISNVRFVIDNDSIKLGTPKVKKLAPLNSASFDLINESKTESKL    180
DSM_7534              NDPYASGYRAKDRLSMKISNVRFVIDNDSIKLGTPKVKKLAPLNSASFDLINESKTESKL    180
DRR016039             NDPYASGYRAKDRLSMKISNVRFVIDNDSIKLGTPKVKKLAPLNSASFDLINESKTESKL    180
VAT12                 NDPYASGYRAKDRLSMKISNVRFVIDNDSIKLGTPKVKKLAPLNSASFDLINESKTESKL    180
RMA8861               NDPYASGYRAKDRLSMKISNVRFVIDNDSIKLGTPKVKKLAPLNSASFDLINESKTESKL    180
MGYG_HGUT_02373       NDPYASGYRAKDRLSMKISNVRFVIDNDSIKLGTPKVKKLAPLNSASFDLINESKTESKL    180
                      ****************:*******************************************

Water_sample          SKTFNYTTSKTVSKTDNFKFGEKIGVKTSFKVGLEAIVDSKVETSFEFNAEQGWSNTNST    240
4015_S_STR            SKTFNYTTSKTVSKTDNFKFGEKIGVKTSFKVGLEAIADSKVETSFEFNAEQGWSNTNST    240
4049_2                SKTFNYTTSKTVSKTDNFKFGEKIGVKTSFKVGLEAIADSKVETSFEFNAEQGWSNTNST    240
WW106                 SKTFNYTTSKTVSKTDNFKFGEKIGVKTSFKVGLEAIADSKVETSFEFNAEQGWSNTNST    240
DSM_7534              SKTFNYTTSKTVSKTDNFKFGEKIGVKTSFKVGLEAIADSKVETSFEFNAEQGWSNTNST    240
DRR016039             SKTFNYTTSKTVSKTDNFKFGEKIGVKTSFKVGLEAIADSKVETSFEFNAEQGWSNTNST    240
VAT12                 SKTFNYTTSKTVSKTDNFKFGEKIGVKTSFKVGLEAIADSKVETSFEFNAEQGWSNTNST    240
RMA8861               SKTFNYTTSKTVSKTDNFKFGEKIGVKTSFKVGLEAIADSKVETSFEFNAEQGWSNTNST    240
MGYG_HGUT_02373       SKTFNYTTSKTVSKTDNFKFGEKIGVKTSFKVGLEAIADSKVETSFEFNAEQGWSNTNST    240
                      *************************************.**********************

Water_sample          TETKQESTTYTATVSPQTKKRLFLDVLGSQIDIPYEGKIYMEYDMELMGFLRYTGNARED    300
4015_S_STR            TETKQESTTYTATVSPQTKKRLFLDVLGSQIDIPYEGKIYMEYDIELMGFLRYTGNARED    300
4049_2                TETKQESTTYTATVSPQTKKRLFLDVLGSQIDIPYEGKIYMEYDIELMGFLRYTGNARED    300
WW106                 TETKQESTTYTATVSPQTKKRLFLDVLGSQIDIPYEGKIYMEYDIELMGFLRYTGNARED    300
DSM_7534              TETKQESTTYTATVSPQTKKRLFLDVLGSQIDIPYEGKIYMEYDIELMGFLRYTGNARED    300
DRR016039             TETKQESTTYTATVSPQTKKRLFLDVLGSQIDIPYEGKIYMEYDIELMGFLRYTGNARED    300
VAT12                 TETKQESTTYTATVSPQTKKRLFLDVLGSQIDIPYEGKIYMEYDIELMGFLRYTGNARED    300
RMA8861               TETKQESTTYTATVSPQTKKRLFLDVLGSQIDIPYEGKIYMEYDIELMGFLRYTGNARED    300
MGYG_HGUT_02373       TETKQESTTYTATVSPQTKKRLFLDVLGSQIDIPYEGKIYMEYDIELMGFLRYTGNARED    300
                      ********************************************:***************
```

```
Water_sample        HTEDRPTVKLKFGKNGMSAEEHLKDLYSHKNINGYSEWDWKWVDEKFGYLFKNSYDALTS     360
4015_S_STR          HTEDRPTVKLKFGKNGMSAEEHLKDLYSHKNINGYSEWDWKWVDEKFGYLFKNSYDALTS     360
4049_2              HTEDRPTVKLKFGKNGMSAEEHLKDLYSHKNINGYSEWDWKWVDEKFGYLFKNSYDALTS     360
WW106               HTEDRPTVKLKFGKNGMSAEEHLKDLYSHKNINGYSEWDWKWVDEKFGYLFKNSYDALTS     360
DSM_7534            HTEDRPTVKLKFGKNGMSAEEHLKDLYSHKNINGYSEWDWKWVDEKFGYLFKNSYDALTS     360
DRR016039           HTEDRPTVKLKFGKNGMSAEEHLKDLYSHKNINGYSEWDWKWVDEKFGYLFKNSYDALTS     360
VAT12               HTEDRPTVKLKFGKNGMSAEEHLKDLYSHKNINGYSEWDWKWVDEKFGYLFKNSYDALTS     360
RMA8861             HTEDRPTVKLKFGKNGMSAEEHLKDLYSHKNINGYSEWDWKWVDEKFGYLFKNSYDALTS     360
MGYG_HGUT_02373     HTEDRPTVKLKFGKNGMSAEEHLKDLYSHKNINGYSEWDWKWVDEKFGYLFKNSYDALTS     360
                    ************************************************************


Water_sample        RKLGGIIKGSFTNINGTKIVIREGKEIPLPDKKRRGKRSVDSLDARLQNEGIRIENIETQ     420
4015_S_STR          RKLGGIIKGSFTNINGTKIVIREGKEIPLPDKKRRGKRSVDSLDARLQNEGIRIENIETQ     420
4049_2              RKLGGIIKGSFTNINGTKIVIREGKEIPLPDKKRRGKRSVDSLDARLQNEGIRIENIETQ     420
WW106               RKLGGIIKGSFTNINGTKIVIREGKEIPLPDKKRRGKRSVDSLDARLQNEGIRIENIETQ     420
DSM_7534            RKLGGIIKGSFTNINGTKIVIREGKEIPLPDKKRRGKRSVDSLDARLQNEGIRIENIETQ     420
DRR016039           RKLGGIIKGSFTNINGTKIVIREGKEIPLPDKKRRGKRSVDSLDARLQNEGIRIENIETQ     420
VAT12               RKLGGIIKGSFTNINGTKIVIREGKEIPLPDKKRRGKRSVDSLDARLQNEGIRIENIETQ     420
RMA8861             RKLGGIIKGSFTNINGTKIVIREGKEIPLPDKKRRGKRSVDSLDARLQNEGIRIENIETQ     420
MGYG_HGUT_02373     RKLGGIIKGSFTNINGTKIVIREGKEIPLPDKKRRGKRSVDSLDARLQNEGIRIENIETQ     420
                    ************************************************************

Water_sample        DVPGFRLNSITYNDKKIDIN**YI   442
4015_S_STR          DVPGFRLNSITYNDKKIDIN**YI   442
4049_2              DVPGFRLNSITYNDKKIDIN**YI   442
WW106               DVPGFRLNSITYNDKKIDIN**YI   442
DSM_7534            DVPGFRLNSITYNDKKIDIN**YI   442
DRR016039           DVPGFRLNSITYNDKKIDIN**YI   442
VAT12               DVPGFRLNSITYNDKKIDIN**YI   442
RMA8861             DVPGFRLNSITYNDKKIDIN**YI   442
MGYG_HGUT_02373     DVPGFRLNSITYNDKKIDIN**YI   442
                    ************************
```

CLUSTAL O(1.2.4) multiple sequence alignment

```
Water_sample        ATGTCAAAAAAATCTTTTGCTAAAAAAGTAATTTGTACATCTATGATTGCAATTCAGTGT	60
DSM_7534            ATGTCAAAAAAATCTTTTGCTAAAAAAGTAATTTGTACATCTATGATTGCAATTCAGTGT	60
VAT12               ATGTCAAAAAAATCTTTTGCTAAAAAAGTAATTTGTACATCTATGATTGCAATTCAGTGT	60
RMA8861             ATGTCAAAAAAATCTTTTGCTAAAAAAGTAATTTGTACATCTATGATTGCAATTCAGTGT	60
4015_S_STR          ATGTCAAAAAAATCTTTTGCTAAAAAAGTAATTTGTACATCTATGATTGCAATTCAGTGT	60
4049_2              ATGTCAAAAAAATCTTTTGCTAAAAAAGTAATTTGTACATCTATGATTGCAATTCAGTGT	60
WW106               ATGTCAAAAAAATCTTTTGCTAAAAAAGTAATTTGTACATCTATGATTGCAATTCAGTGT	60
DRR016039           ATGTCAAAAAAATCTTTTGCTAAAAAAGTAATTTGTACATCTATGATTGCAATTCAGTGT	60
MGYG_HGUT_02373     ATGTCAAAAAAATCTTTTGCTAAAAAAGTAATTTGTACATCTATGATTGCAATTCAGTGT	60
                    ************************************************************

Water_sample        GCGGCAGTAGTACCACATGTACAAGCTTATGCACTTACAAATTTTGAAGAGGGGGGATAT	120
DSM_7534            GCGGCAGTAGTACCACATGTACAAGCTTATGCACTTACAAATCTTGAAGAGGGGGGATAT	120
VAT12               GCGGCAGTAGTACCACATGTACAAGCTTATGCACTTACAAATCTTGAAGAGGGGGGATAT	120
RMA8861             GCGGCAGTAGTACCACATGTACAAGCTTATGCACTTACAAATCTTGAAGAGGGGGGATAT	120
4015_S_STR          GCGGCAGTAGTACCACATGTACAAGCTTATGCACTTACAAATCTTGAAGAGGGGGGATAT	120
4049_2              GCGGCAGTAGTACCACATGTACAAGCTTATGCACTTACAAATCTTGAAGAGGGGGGATAT	120
WW106               GCGGCAGTAGTACCACATGTACAAGCTTATGCACTTACAAATCTTGAAGAGGGGGGATAT	120
DRR016039           GCGGCAGTAGTACCACATGTACAAGCTTATGCACTTACAAATCTTGAAGAGGGGGGATAT	120
MGYG_HGUT_02373     GCGGCAGTAGTACCACATGTACAAGCTTATGCACTTACAAATCTTGAAGAGGGGGGATAT	120
                    ****************************************** *****************

Water_sample        GCAAATCATAATAATGCTTCTTCAATTAAAATATTTGGATATGAAGACAATGAAGATTTA	180
DSM_7534            GCAAATCATAATAATGCTTCTTCAATTAAAATATTTGGATATGAAGACAATGAAGATTTA	180
VAT12               GCAAATCATAATAATGCTTCTTCAATTAAAATATTTGGATATGAAGACAATGAAGATTTA	180
RMA8861             GCAAATCATAATAATGCTTCTTCAATTAAAATATTTGGATATGAAGACAATGAAGATTTA	180
4015_S_STR          GCAAATCATAATAATGCTTCTTCAATTAAAATATTTGGATATGAAGACAATGAAGATTTA	180
4049_2              GCAAATCATAATAATGCTTCTTCAATTAAAATATTTGGATATGAAGACAATGAAGATTTA	180
WW106               GCAAATCATAATAATGCTTCTTCAATTAAAATATTTGGATATGAAGACAATGAAGATTTA	180
DRR016039           GCAAATCATAATAATGCTTCTTCAATTAAAATATTTGGATATGAAGACAATGAAGATTTA	180
MGYG_HGUT_02373     GCAAATCATAATAATGCTTCTTCAATTAAAATATTTGGATATGAAGACAATGAAGATTTA	180
                    ************************************************************

Water_sample        AAAGCTAAAATTATTCAAGATCCAGAGTTTATAAGAAATTGGGCAAATGTAGCTCATTCA	240
DSM_7534            AAAGCTAAAATTATTCAAGATCCAGAGTTTATAAGAAATTGGGCAAATGTAGCTCATTCA	240
VAT12               AAAGCTAAAATTATTCAAGATCCAGAGTTTATAAGAAATTGGGCAAATGTAGCTCATTCA	240
RMA8861             AAAGCTAAAATTATTCAAGATCCAGAGTTTATAAGAAATTGGGCAAATGTAGCTCATTCA	240
4015_S_STR          AAAGCTAAAATTATTCAAGATCCAGAGTTTATAAGAAATTGGGCAAATGTAGCTCATTCA	240
4049_2              AAAGCTAAAATTATTCAAGATCCAGAGTTTATAAGAAATTGGGCAAATGTAGCTCATTCA	240
WW106               AAAGCTAAAATTATTCAAGATCCAGAGTTTATAAGAAATTGGGCAAATGTAGCTCATTCA	240
DRR016039           AAAGCTAAAATTATTCAAGATCCAGAGTTTATAAGAAATTGGGCAAATGTAGCTCATTCA	240
MGYG_HGUT_02373     AAAGCTAAAATTATTCAAGATCCAGAGTTTATAAGAAATTGGGCAAATGTAGCTCATTCA	240
                    ************************************************************

Water_sample        TTAGGATTTGGATGGTGCGGTGGAACGGCTAATCCAAACGTTGGACAAGGTTTTGAATTT	300
DSM_7534            TTAGGATTTGGATGGTGCGGTGGAACGGCTAATCCAAATGTTGGACAAGGTTTTGAATTT	300
VAT12               TTAGGATTTGGATGGTGCGGTGGAACGGCTAATCCAAATGTTGGACAAGGTTTTGAATTT	300
RMA8861             TTAGGATTTGGATGGTGCGGTGGAACGGCTAATCCAAATGTTGGACAAGGTTTTGAATTT	300
4015_S_STR          TTAGGATTTGGATGGTGCGGTGGAACGGCTAATCCAAACGTTGGACAAGGTTTTGAATTT	300
4049_2              TTAGGATTTGGATGGTGCGGTGGAACGGCTAATCCAAACGTTGGACAAGGTTTTGAATTT	300
WW106               TTAGGATTTGGATGGTGCGGTGGAACGGCTAATCCAAACGTTGGACAAGGTTTTGAATTT	300
DRR016039           TTAGGATTTGGATGGTGCGGTGGAACGGCTAATCCAAACGTTGGACAAGGTTTTGAATTT	300
MGYG_HGUT_02373     TTAGGATTTGGATGGTGCGGTGGAACGGCTAATCCAAACGTTGGACAAGGTTTTGAATTT	300
                    ************************************** *********************

Water_sample        AAAAGAGAAGTTGGGGCAGGTGGAAAAGTATCTTATTTATTATCTGCTAGATACAATCCA	360
DSM_7534            AAAAGAGAAGTTGGGGCAGGTGGAAAAGTATCTTATTTATTATCTGCTAGATACAATCCA	360
VAT12               AAAAGAGAAGTTGGGGCAGGTGGAAAAGTATCTTATTTATTATCTGCTAGATACAATCCA	360
RMA8861             AAAAGAGAAGTTGGGGCAGGTGGAAAAGTATCTTATTTATTATCTGCTAGATACAATCCA	360
4015_S_STR          AAAAGAGAAGTTGGGGCAGGTGGAAAAGTATCTTATTTATTATCTGCTAGATACAATCCA	360
4049_2              AAAAGAGAAGTTGGGGCAGGTGGAAAAGTATCTTATTTATTATCTGCTAGATACAATCCA	360
WW106               AAAAGAGAAGTTGGGGCAGGTGGAAAAGTATCTTATTTATTATCTGCTAGATACAATCCA	360
DRR016039           AAAAGAGAAGTTGGGGCAGGTGGAAAAGTATCTTATTTATTATCTGCTAGATACAATCCA	360
MGYG_HGUT_02373     AAAAGAGAAGTTGGGGCAGGTGGAAAAGTATCTTATTTATTATCTGCTAGATACAATCCA	360
                    ************************************************************
```

```
Water_sample        AATGATCCTTATGCAAGTGGATATCGTGCAAAAGATAGACTGTCTATGAGAATATCAAAT     420
DSM_7534            AATGATCCTTATGCAAGTGGGTATCGTGCAAAAGATAGACTTTCTATGAAAATATCAAAT     420
VAT12               AATGATCCTTATGCAAGTGGGTATCGTGCAAAAGATAGACTTTCTATGAAAATATCAAAT     420
RMA8861             AATGATCCTTATGCAAGTGGGTATCGTGCAAAAGATAGACTTTCTATGAAAATATCAAAT     420
4015_S_STR          AATGATCCTTATGCAAGTGGATATCGTGCAAAAGATAGACTTTCTATGAAAATATCAAAT     420
4049_2              AATGATCCTTATGCAAGTGGATATCGTGCAAAAGATAGACTTTCTATGAAAATATCAAAT     420
WW106               AATGATCCTTATGCAAGTGGGTATCGTGCAAAAGATAGACTTTCTATGAAAATATCAAAT     420
DRR016039           AATGATCCTTATGCAAGTGGGTATCGTGCAAAAGATAGACTTTCTATGAAAATATCAAAT     420
MGYG_HGUT_02373     AATGATCCTTATGCAAGTGGGTATCGTGCAAAAGATAGACTTTCTATGAAAATATCAAAT     420
                    ******************** ******************** ******* **********

Water_sample        GTTAGATTTGTTATTGATAATGATTCTATAAAATTAGGTACACCTAAAGTGAAAAAATTA     480
DSM_7534            GTTAGATTTGTTATTGATAATGATTCTATAAAATTAGGTACACCTAAAGTGAAAAAATTA     480
VAT12               GTTAGATTTGTTATTGATAATGATTCTATAAAATTAGGTACACCTAAAGTGAAAAAATTA     480
RMA8861             GTTAGATTTGTTATTGATAATGATTCTATAAAATTAGGTACACCTAAAGTGAAAAAATTA     480
4015_S_STR          GTTAGATTTGTTATTGATAATGATTCTATAAAATTAGGTACACCTAAAGTGAAAAAATTA     480
4049_2              GTTAGATTTGTTATTGATAATGATTCTATAAAATTAGGTACACCTAAAGTGAAAAAATTA     480
WW106               GTTAGATTTGTTATTGATAATGATTCTATAAAATTAGGTACACCTAAAGTGAAAAAATTA     480
DRR016039           GTTAGATTTGTTATTGATAATGATTCTATAAAATTAGGTACACCTAAAGTGAAAAAATTA     480
MGYG_HGUT_02373     GTTAGATTTGTTATTGATAATGATTCTATAAAATTAGGTACACCTAAAGTGAAAAAATTA     480
                    ************************************************************

Water_sample        GCACCTTTAAACTCTGCTAGTTTTGATTTAATAAATGAAAGTAAAACTGAGTCTAAATTA     540
DSM_7534            GCACCTTTAAACTCTGCTAGTTTTGATTTAATAAATGAAAGTAAAACTGAGTCTAAATTA     540
VAT12               GCACCTTTAAACTCTGCTAGTTTTGATTTAATAAATGAAAGTAAAACTGAGTCTAAATTA     540
RMA8861             GCACCTTTAAACTCTGCTAGTTTTGATTTAATAAATGAAAGTAAAACTGAGTCTAAATTA     540
4015_S_STR          GCACCTTTAAACTCTGCTAGTTTTGATTTAATAAATGAAAGTAAAACTGAGTCTAAATTA     540
4049_2              GCACCTTTAAACTCTGCTAGTTTTGATTTAATAAATGAAAGTAAAACTGAGTCTAAATTA     540
WW106               GCACCTTTAAACTCTGCTAGTTTTGATTTAATAAATGAAAGTAAAACTGAGTCTAAATTA     540
DRR016039           GCACCTTTAAACTCTGCTAGTTTTGATTTAATAAATGAAAGTAAAACTGAGTCTAAATTA     540
MGYG_HGUT_02373     GCACCTTTAAACTCTGCTAGTTTTGATTTAATAAATGAAAGTAAAACTGAGTCTAAATTA     540
                    ************************************************************

Water_sample        TCAAAAACATTTAATTATACAACTTCTAAAACAGTTTCTAAAACAGATAACTTTAAATTT     600
DSM_7534            TCAAAAACATTTAATTATACAACTTCTAAAACAGTTTCTAAAACAGATAACTTTAAATTT     600
VAT12               TCAAAAACATTTAATTATACAACTTCTAAAACAGTTTCTAAAACAGATAACTTTAAATTT     600
RMA8861             TCAAAAACATTTAATTATACAACTTCTAAAACAGTTTCTAAAACAGATAACTTTAAATTT     600
4015_S_STR          TCAAAAACATTTAATTATACAACTTCTAAAACAGTTTCTAAAACAGATAACTTTAAATTT     600
4049_2              TCAAAAACATTTAATTATACAACTTCTAAAACAGTTTCTAAAACAGATAACTTTAAATTT     600
WW106               TCAAAAACATTTAATTATACAACTTCTAAAACAGTTTCTAAAACAGATAACTTTAAATTT     600
DRR016039           TCAAAAACATTTAATTATACAACTTCTAAAACAGTTTCTAAAACAGATAACTTTAAATTT     600
MGYG_HGUT_02373     TCAAAAACATTTAATTATACAACTTCTAAAACAGTTTCTAAAACAGATAACTTTAAATTT     600
                    ************************************************************

Water_sample        GGAGAAAAAATAGGAGTAAAAACATCATTTAAAGTAGGTCTTGAAGCTATAGTTGACAGT     660
DSM_7534            GGAGAAAAAATAGGAGTAAAAACATCATTTAAAGTAGGTCTTGAAGCTATAGCTGACAGT     660
VAT12               GGAGAAAAAATAGGAGTAAAAACATCATTTAAAGTAGGTCTTGAAGCTATAGCTGACAGT     660
RMA8861             GGAGAAAAAATAGGAGTAAAAACATCATTTAAAGTAGGTCTTGAAGCTATAGCTGACAGT     660
4015_S_STR          GGAGAAAAAATAGGAGTAAAAACATCATTTAAAGTAGGTCTTGAAGCTATAGCTGACAGT     660
4049_2              GGAGAAAAAATAGGAGTAAAAACATCATTTAAAGTAGGTCTTGAAGCTATAGCTGACAGT     660
WW106               GGAGAAAAAATAGGAGTAAAAACATCATTTAAAGTAGGTCTTGAAGCTATAGCTGACAGT     660
DRR016039           GGAGAAAAAATAGGAGTAAAAACATCATTTAAAGTAGGTCTTGAAGCTATAGCTGACAGT     660
MGYG_HGUT_02373     GGAGAAAAAATAGGAGTAAAAACATCATTTAAAGTAGGTCTTGAAGCTATAGCTGACAGT     660
                    **************************************************** *******

Water_sample        AAAGTTGAGACAAGCTTTGAATTTAATGCAGAACAAGGTTGGTCAAATACAAATAGTACT     720
DSM_7534            AAAGTTGAGACAAGCTTTGAATTTAATGCAGAACAAGGTTGGTCAAATACAAATAGTACT     720
VAT12               AAAGTTGAGACAAGCTTTGAATTTAATGCAGAACAAGGTTGGTCAAATACAAATAGTACT     720
RMA8861             AAAGTTGAGACAAGCTTTGAATTTAATGCAGAACAAGGTTGGTCAAATACAAATAGTACT     720
4015_S_STR          AAAGTTGAGACAAGCTTTGAATTTAATGCAGAACAAGGTTGGTCAAATACAAATAGTACT     720
4049_2              AAAGTTGAGACAAGCTTTGAATTTAATGCAGAACAAGGTTGGTCAAATACAAATAGTACT     720
WW106               AAAGTTGAGACAAGCTTTGAATTTAATGCAGAACAAGGTTGGTCAAATACAAATAGTACT     720
DRR016039           AAAGTTGAGACAAGCTTTGAATTTAATGCAGAACAAGGTTGGTCAAATACAAATAGTACT     720
MGYG_HGUT_02373     AAAGTTGAGACAAGCTTTGAATTTAATGCAGAACAAGGTTGGTCAAATACAAATAGTACT     720
                    ************************************************************
```

```
Water_sample        ACTGAAACTAAACAAGAAAGTACTACATATACTGCAACAGTTTCTCCACAAACTAAAAAG    780
DSM_7534            ACTGAAACTAAACAAGAAAGTACTACATATACTGCAACAGTTTCTCCACAAACTAAAAAG    780
VAT12               ACTGAAACTAAACAAGAAAGTACTACATATACTGCAACAGTTTCTCCACAAACTAAAAAG    780
RMA8861             ACTGAAACTAAACAAGAAAGTACTACATATACTGCAACAGTTTCTCCACAAACTAAAAAG    780
4015_S_STR          ACTGAAACTAAACAAGAAAGTACTACATATACTGCAACAGTTTCTCCACAAACTAAAAAG    780
4049_2              ACTGAAACTAAACAAGAAAGTACTACATATACTGCAACAGTTTCTCCACAAACTAAAAAG    780
WW106               ACTGAAACTAAACAAGAAAGTACTACATATACTGCAACAGTTTCTCCACAAACTAAAAAG    780
DRR016039           ACTGAAACTAAACAAGAAAGTACTACATATACTGCAACAGTTTCTCCACAAACTAAAAAG    780
MGYG_HGUT_02373     ACTGAAACTAAACAAGAAAGTACTACATATACTGCAACAGTTTCTCCACAAACTAAAAAG    780
                    ************************************************************

Water_sample        AGATTATTCCTAGATGTGTTAGGATCACAAATTGATATTCCTTATGAAGGAAAAATATAT    840
DSM_7534            AGATTATTCCTAGATGTGTTAGGATCACAAATTGATATTCCTTATGAAGGAAAAATATAT    840
VAT12               AGATTATTCCTAGATGTGTTAGGATCACAAATTGATATTCCTTATGAAGGAAAAATATAT    840
RMA8861             AGATTATTCCTAGATGTGTTAGGATCACAAATTGATATTCCTTATGAAGGAAAAATATAT    840
4015_S_STR          AGATTATTCCTAGATGTGTTAGGATCACAAATTGATATTCCTTATGAAGGAAAAATATAT    840
4049_2              AGATTATTCCTAGATGTGTTAGGATCACAAATTGATATTCCTTATGAAGGAAAAATATAT    840
WW106               AGATTATTCCTAGATGTGTTAGGATCACAAATTGATATTCCTTATGAAGGAAAAATATAT    840
DRR016039           AGATTATTCCTAGATGTGTTAGGATCACAAATTGATATTCCTTATGAAGGAAAAATATAT    840
MGYG_HGUT_02373     AGATTATTCCTAGATGTGTTAGGATCACAAATTGATATTCCTTATGAAGGAAAAATATAT    840
                    ************************************************************

Water_sample        ATGGAATACGACATGGAATTAATGGGATTTTTAAGATATACAGGAAATGCTCGTGAAGAT    900
DSM_7534            ATGGAATACGACATAGAATTAATGGGATTTTTAAGATATACAGGAAATGCTCGTGAAGAT    900
VAT12               ATGGAATACGACATAGAATTAATGGGATTTTTAAGATATACAGGAAATGCTCGTGAAGAT    900
RMA8861             ATGGAATACGACATAGAATTAATGGGATTTTTAAGATATACAGGAAATGCTCGTGAAGAT    900
4015_S_STR          ATGGAATACGACATAGAATTAATGGGATTTTTAAGATATACAGGAAATGCTCGTGAAGAT    900
4049_2              ATGGAATACGACATAGAATTAATGGGATTTTTAAGATATACAGGAAATGCTCGTGAAGAT    900
WW106               ATGGAATACGACATAGAATTAATGGGATTTTTAAGATATACAGGAAATGCTCGTGAAGAT    900
DRR016039           ATGGAATACGACATAGAATTAATGGGATTTTTAAGATATACAGGAAATGCTCGTGAAGAT    900
MGYG_HGUT_02373     ATGGAATACGACATAGAATTAATGGGATTTTTAAGATATACAGGAAATGCTCGTGAAGAT    900
                    ************** *********************************************

Water_sample        CATACTGAAGATAGACCAACAGTTAAACTTAAATTTGGTAAAAACGGTATGAGTGCTGAG    960
DSM_7534            CATACTGAAGATAGACCAACAGTTAAACTTAAATTTGGTAAAAACGGTATGAGTGCTGAG    960
VAT12               CATACTGAAGATAGACCAACAGTTAAACTTAAATTTGGTAAAAACGGTATGAGTGCTGAG    960
RMA8861             CATACTGAAGATAGACCAACAGTTAAACTTAAATTTGGTAAAAACGGTATGAGTGCTGAG    960
4015_S_STR          CATACTGAAGATAGACCAACAGTTAAACTTAAATTTGGTAAAAACGGTATGAGTGCTGAG    960
4049_2              CATACTGAAGATAGACCAACAGTTAAACTTAAATTTGGTAAAAACGGTATGAGTGCTGAG    960
WW106               CATACTGAAGATAGACCAACAGTTAAACTTAAATTTGGTAAAAACGGTATGAGTGCTGAG    960
DRR016039           CATACTGAAGATAGACCAACAGTTAAACTTAAATTTGGTAAAAACGGTATGAGTGCTGAG    960
MGYG_HGUT_02373     CATACTGAAGATAGACCAACAGTTAAACTTAAATTTGGTAAAAACGGTATGAGTGCTGAG    960
                    ************************************************************

Water_sample        GAACATCTTAAAGATTTATATAGTCATAAGAATATTAATGGATATTCAGAATGGGATTGG    1020
DSM_7534            GAACATCTTAAAGATTTATATAGTCATAAGAATATTAATGGATATTCAGAATGGGATTGG    1020
VAT12               GAACATCTTAAAGATTTATATAGTCATAAGAATATTAATGGATATTCAGAATGGGATTGG    1020
RMA8861             GAACATCTTAAAGATTTATATAGTCATAAGAATATTAATGGATATTCAGAATGGGATTGG    1020
4015_S_STR          GAACATCTTAAAGATTTATATAGTCATAAGAATATTAATGGATATTCAGAATGGGATTGG    1020
4049_2              GAACATCTTAAAGATTTATATAGTCATAAGAATATTAATGGATATTCAGAATGGGATTGG    1020
WW106               GAACATCTTAAAGATTTATATAGTCATAAGAATATTAATGGATATTCAGAATGGGATTGG    1020
DRR016039           GAACATCTTAAAGATTTATATAGTCATAAGAATATTAATGGATATTCAGAATGGGATTGG    1020
MGYG_HGUT_02373     GAACATCTTAAAGATTTATATAGTCATAAGAATATTAATGGATATTCAGAATGGGATTGG    1020
                    ************************************************************

Water_sample        AAATGGGTAGATGAGAAATTTGGTTATTTATTTAAAAATTCATACGATGCTCTTACTAGT    1080
DSM_7534            AAATGGGTAGATGAGAAATTTGGTTATTTATTTAAAAATTCATACGATGCTCTTACTAGT    1080
VAT12               AAATGGGTAGATGAGAAATTTGGTTATTTATTTAAAAATTCATACGATGCTCTTACTAGT    1080
RMA8861             AAATGGGTAGATGAGAAATTTGGTTATTTATTTAAAAATTCATACGATGCTCTTACTAGT    1080
4015_S_STR          AAATGGGTAGATGAGAAATTTGGTTATTTATTTAAAAATTCATACGATGCTCTTACTAGT    1080
4049_2              AAATGGGTAGATGAGAAATTTGGTTATTTATTTAAAAATTCATACGATGCTCTTACTAGT    1080
WW106               AAATGGGTAGATGAGAAATTTGGTTATTTATTTAAAAATTCATACGATGCTCTTACTAGT    1080
DRR016039           AAATGGGTAGATGAGAAATTTGGTTATTTATTTAAAAATTCATACGATGCTCTTACTAGT    1080
MGYG_HGUT_02373     AAATGGGTAGATGAGAAATTTGGTTATTTATTTAAAAATTCATACGATGCTCTTACTAGT    1080
                    ************************************************************
```

```
Water_sample        AGAAAATTAGGAGGAATAATAAAAGGCTCATTTACCAACATTAATGGAACAAAAATAGTA    1140
DSM_7534            AGAAAATTAGGAGGAATAATAAAAGGCTCATTTACTAACATTAATGGAACAAAAATAGTA    1140
VAT12               AGAAAATTAGGAGGAATAATAAAAGGCTCATTTACTAACATTAATGGAACAAAAATAGTA    1140
RMA8861             AGAAAATTAGGAGGAATAATAAAAGGCTCATTTACTAACATTAATGGAACAAAAATAGTA    1140
4015_S_STR          AGAAAATTAGGAGGAATAATAAAAGGCTCATTTACTAACATTAATGGAACAAAAATAGTA    1140
4049_2              AGAAAATTAGGAGGAATAATAAAAGGCTCATTTACTAACATTAATGGAACAAAAATAGTA    1140
WW106               AGAAAATTAGGAGGAATAATAAAAGGCTCATTTACTAACATTAATGGAACAAAAATAGTA    1140
DRR016039           AGAAAATTAGGAGGAATAATAAAAGGCTCATTTACTAACATTAATGGAACAAAAATAGTA    1140
MGYG_HGUT_02373     AGAAAATTAGGAGGAATAATAAAAGGCTCATTTACTAACATTAATGGAACAAAAATAGTA    1140
                    *********************************** ************************

Water_sample        ATTAGAGAAGGTAAAGAAATTCCACTTCCTGATAAGAAGAGAAGAGGAAAACGTTCAGTA    1200
DSM_7534            ATTAGAGAAGGTAAAGAAATTCCACTTCCTGATAAGAAGAGAAGAGGAAAACGTTCAGTA    1200
VAT12               ATTAGAGAAGGTAAAGAAATTCCACTTCCTGATAAGAAGAGAAGAGGAAAACGTTCAGTA    1200
RMA8861             ATTAGAGAAGGTAAAGAAATTCCACTTCCTGATAAGAAGAGAAGAGGAAAACGTTCAGTA    1200
4015_S_STR          ATTAGAGAAGGTAAAGAAATTCCACTTCCTGATAAGAAGAGAAGAGGAAAACGTTCAGTA    1200
4049_2              ATTAGAGAAGGTAAAGAAATTCCACTTCCTGATAAGAAGAGAAGAGGAAAACGTTCAGTA    1200
WW106               ATTAGAGAAGGTAAAGAAATTCCACTTCCTGATAAGAAGAGAAGAGGAAAACGTTCAGTA    1200
DRR016039           ATTAGAGAAGGTAAAGAAATTCCACTTCCTGATAAGAAGAGAAGAGGAAAACGTTCAGTA    1200
MGYG_HGUT_02373     ATTAGAGAAGGTAAAGAAATTCCACTTCCTGATAAGAAGAGAAGAGGAAAACGTTCAGTA    1200
                    ************************************************************

Water_sample        GATTCTTTAGATGCTAGATTACAAAATGAAGGTATTAGAATAGAAAATATTGAAACACAA    1260
DSM_7534            GATTCTTTAGATGCTAGATTACAAAATGAAGGTATTAGAATAGAAAATATTGAAACACAA    1260
VAT12               GATTCTTTAGATGCTAGATTACAAAATGAAGGTATTAGAATAGAAAATATTGAAACACAA    1260
RMA8861             GATTCTTTAGATGCTAGATTACAAAATGAAGGTATTAGAATAGAAAATATTGAAACACAA    1260
4015_S_STR          GATTCTTTAGATGCTAGATTACAAAATGAAGGTATTAGAATAGAAAATATTGAAACACAA    1260
4049_2              GATTCTTTAGATGCTAGATTACAAAATGAAGGTATTAGAATAGAAAATATTGAAACACAA    1260
WW106               GATTCTTTAGATGCTAGATTACAAAATGAAGGTATTAGAATAGAAAATATTGAAACACAA    1260
DRR016039           GATTCTTTAGATGCTAGATTACAAAATGAAGGTATTAGAATAGAAAATATTGAAACACAA    1260
MGYG_HGUT_02373     GATTCTTTAGATGCTAGATTACAAAATGAAGGTATTAGAATAGAAAATATTGAAACACAA    1260
                    ************************************************************

Water_sample        GATGTTCCAGGATTTAGACTAAATAGCATAACATACAATGATAAAAAAATTGATATTAAT    1320
DSM_7534            GATGTTCCAGGATTTAGACTAAATAGCATAACATACAATGATAAAAAAATTGATATTAAT    1320
VAT12               GATGTTCCAGGATTTAGACTAAATAGCATAACATACAATGATAAAAAAATTGATATTAAT    1320
RMA8861             GATGTTCCAGGATTTAGACTAAATAGCATAACATACAATGATAAAAAAATTGATATTAAT    1320
4015_S_STR          GATGTTCCAGGATTTAGACTAAATAGCATAACATACAATGATAAAAAAATTGATATTAAT    1320
4049_2              GATGTTCCAGGATTTAGACTAAATAGCATAACATACAATGATAAAAAAATTGATATTAAT    1320
WW106               GATGTTCCAGGATTTAGACTAAATAGCATAACATACAATGATAAAAAAATTGATATTAAT    1320
DRR016039           GATGTTCCAGGATTTAGACTAAATAGCATAACATACAATGATAAAAAAATTGATATTAAT    1320
MGYG_HGUT_02373     GATGTTCCAGGATTTAGACTAAATAGCATAACATACAATGATAAAAAAATTGATATTAAT    1320
                    ************************************************************

Water_sample        TAATAATATATAA   1333
DSM_7534            TAATAATATATAA   1333
VAT12               TAATAATATATAA   1333
RMA8861             TAATAATATATAA   1333
4015_S_STR          TAATAATATATAA   1333
4049_2              TAATAATATATAA   1333
WW106               TAATAATATATAA   1333
DRR016039           TAATAATATATAA   1333
MGYG_HGUT_02373     TAATAATATATAA   1333
                    *************
```

References

1. Madeira F, Pearce M, Tivey ARN, Basutkar P, Lee J, Edbali O, et al. Search and sequence analysis tools services from EMBL-EBI in 2022. Nucleic Acids Res. 2022;50(W1):W276-W9.
