# Supplementary material for: Severe subcutaneous infection with Clostridium septicum in a herd of native Icelandic horses
Source: Acta Vet Scand. 2025 Feb 6;67:8. doi: 10.1186/s13028-025-00792-y (PMC11800538; doi:10.1186/s13028-025-00792-y)
Supplement: Supplementary file 6 — Additional file 6. A summary of the results from the ClonalFrameML analysis of recombination in the genome assemblies for all of the Clostridium septicum strains. [file 13028_2025_792_MOESM6_ESM.pdf]

Additional file 6. A summary of the results from ClonalFrameML analysis of recombination in the genome assemblies for all the C. septicum strains. The figure is an output from the program cfml_results.R and shows the results from ClonalFrameML (1) analysis of recombination in the genome assemblies for all the C. septicum strains. On the left of the image is a tree that shows the evolutionary relationship of the strains according to

ClonalFrameML having considered detected recombination events. Recombination events on the right of the image are indicated with dark blue. Light blue indicates no change, whereas a color gradient from white to yellow indicates non-homoplastic to homoplastic changes, the stronger the color the higher the degree of homoplasy.

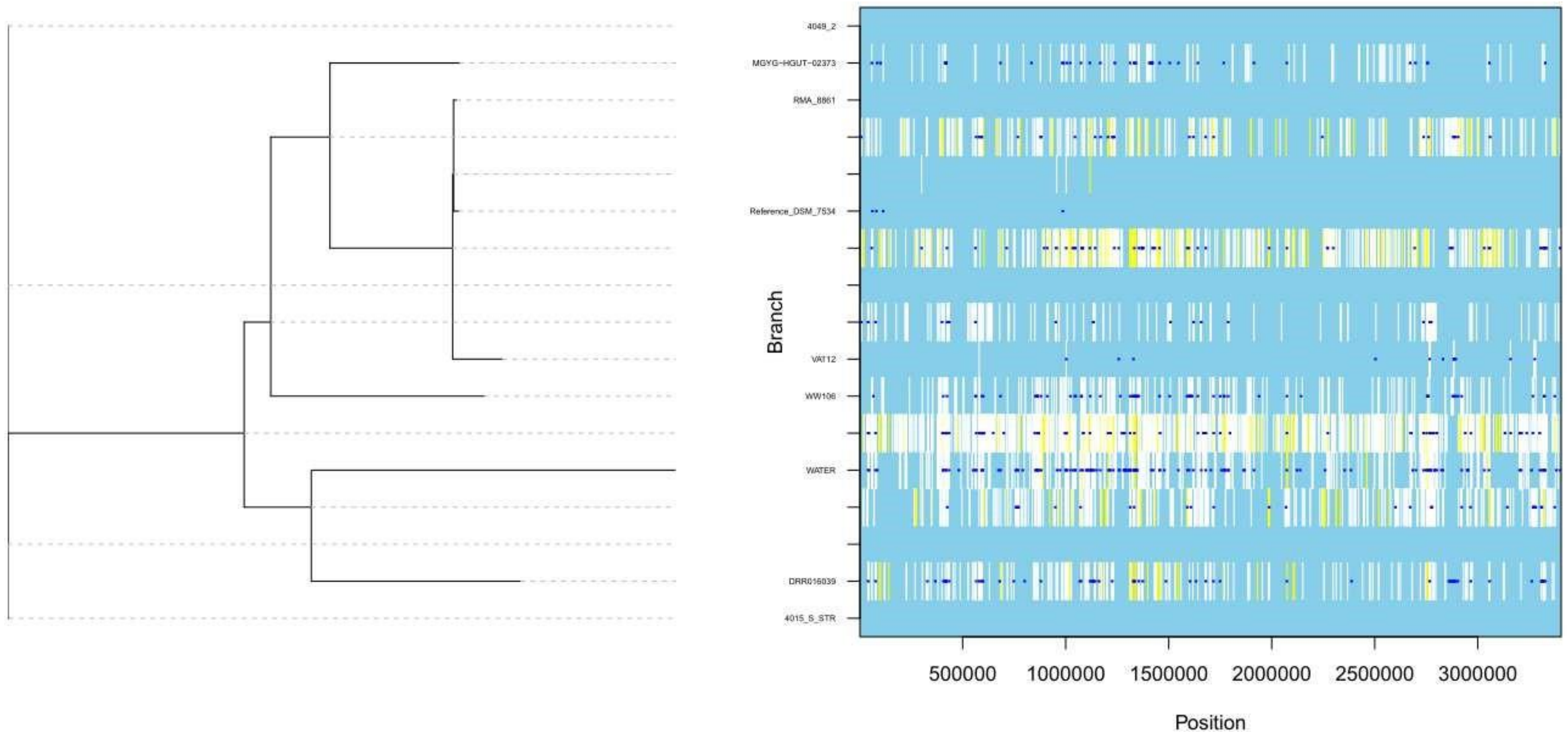

References

1. Didelot X, Wilson DJ. ClonalFrameML: Efficient Inference of Recombination in Whole Bacterial Genomes. Plos Comput Biol. 2015;11(2).
